# Convergence rates of Gaussian ODE filters

**DOI:** 10.1007/s11222-020-09972-4

**Published:** 2020-09-12

**Authors:** Hans Kersting, T. J. Sullivan, Philipp Hennig

**Affiliations:** 1 University of Tübingen and Max Planck Institute for Intelligent Systems, Maria-von-Linden-Straße 6, 72076 Tübingen, Germany; 2 University of Warwick, Coventry, CV4 7AL United Kingdom; 3 Zuse Institute Berlin, Takustraße 7, 14195 Berlin, Germany

**Keywords:** Probabilistic numerics, Ordinary differential equations, Initial value problems, Numerical analysis, Gaussian processes, Markov processes, 65L20, 37H10, 68W20, 93E11

## Abstract

A recently introduced class of probabilistic (uncertainty-aware) solvers for ordinary differential equations (ODEs) applies Gaussian (Kalman) filtering to initial value problems. These methods model the true solution *x* and its first *q* derivatives *a priori* as a Gauss–Markov process ${\varvec{X}}$, which is then iteratively conditioned on information about ${\dot{x}}$. This article establishes worst-case local convergence rates of order $q+1$ for a wide range of versions of this Gaussian ODE filter, as well as global convergence rates of order *q* in the case of $q=1$ and an integrated Brownian motion prior, and analyses how inaccurate information on ${\dot{x}}$ coming from approximate evaluations of *f* affects these rates. Moreover, we show that, in the globally convergent case, the posterior credible intervals are well calibrated in the sense that they globally contract at the same rate as the truncation error. We illustrate these theoretical results by numerical experiments which might indicate their generalizability to $q \in \{2,3,\ldots \}$.

## Introduction

A solver of an initial value problem (IVP) outputs an approximate solution ${\hat{x}} :[0,T] \rightarrow {\mathbb {R}}^d$ of an ordinary differential equation (ODE) with initial condition:

1 $$\begin{aligned} x^{(1)}(t) :=\frac{\mathrm {d}x}{\mathrm {d}t} (t)&= f \left( x(t) \right) ,\qquad \forall t\in [0,T], \nonumber \\ x(0)&= x_0\ \in {\mathbb {R}}^d. \end{aligned}$$

(Without loss of generality, we simplify the presentation by restricting attention to the autonomous case.) The numerical solution ${\hat{x}}$ is computed by iteratively collecting information on $x^{(1)}(t)$ by evaluating $f :{\mathbb {R}}^d \rightarrow {\mathbb {R}}^d$ at a numerical estimate ${\hat{x}}(t)$ of *x*(*t*) and using these approximate evaluations of the time derivative to extrapolate along the time axis. In other words, the numerical solution (or *estimator*) ${\hat{x}}$ of the exact solution (or *estimand*) *x* is calculated based on evaluations of the vector field *f* (or *data*). Accordingly, we treat ${\hat{x}}$ itself as an estimator, i.e., a statistic that translates evaluations of *f* into a probability distribution over $C^1 ([0,T];{\mathbb {R}}^d)$, the space of continuously differentiable functions from [0, *T*] to ${\mathbb {R}}^d$.

This probabilistic interpretation of numerical computations of tractable from intractable quantities as statistical inference of latent from observable quantities applies to all numerical problems and has been repeatedly recommended in the past (Poincaré 1896; Diaconis 1988; Skilling 1991; O’Hagan 1992; Ritter 2000). It employs the language of probability theory to account for the epistemic uncertainty (i.e., limited knowledge) about the accuracy of intermediate and final numerical computations, thereby yielding algorithms which can be more aware of—as well as more robust against—uncertainty over intermediate computational results. Such algorithms can output probability measures, instead of point estimates, over the final quantity of interest. This approach, now called *probabilistic numerics (PN)* (Hennig et al. 2015; Oates and Sullivan 2019), has in recent years been spelled out for a wide range of numerical tasks, including linear algebra, optimization, integration, and differential equations, thereby working towards the long-term goal of a coherent framework to propagate uncertainty through chained computations, as desirable, e.g., in statistical machine learning.

In this paper, we determine the convergence rates of a recent family of PN methods (Schober et al. 2014; Kersting and Hennig 2016; Magnani et al. 2017; Schober et al. 2019; Tronarp et al. 2019) which recast an IVP as a *stochastic filtering problem* (Øksendal 2003, Chapter 6), an approach that has been studied in other settings (Jazwinski 1970), but has not been applied to IVPs before. These methods assume *a priori* that the solution *x* and its first $q \in {\mathbb {N}}$ derivatives follow a Gauss–Markov process ${\varvec{X}}$ that solves a stochastic differential equation (SDE).

The evaluations of *f* at numerical estimates of the true solution can then be regarded as imperfect evaluations of ${\dot{x}}$, which can then be used for a Bayesian update of ${\varvec{X}}$. Such recursive updates along the time axis yield an algorithm whose structure resembles that of Gaussian (Kalman) filtering (Särkkä 2013, Chapter 4). These methods add only slight computational overhead compared to classical methods (Schober et al. 2019) and have been shown to inherit local convergence rates from equivalent classical methods in specific cases (Schober et al. 2014, 2019). These equivalences (i.e., the equality of the filtering posterior mean and the classical method) are only known to hold in the case of the integrated Brownian motion (IBM) prior and noiseless evaluations of *f* (in terms of our later notation, the case $R\equiv 0$), as well as under the following restrictions:

Firstly, for $q \in \{1,2,3\}$, and if the first step is divided into sub-steps resembling those of Runge–Kutta methods, an equivalence of the posterior mean of the first step of the filter and the explicit Runge–Kutta method of order *q* was established in Schober et al. (2014) (but for $q \in \{2,3\}$ only in the limit as the initial time of the IBM tends to $-\infty$). Secondly, it was shown by Schober et al. (2019) that, for $q=1$, the posterior mean after each step coincides with the trapezoidal rule if it takes an additional evaluation of *f* at the end of each step, known as P(EC)1. The same paper shows that, for $q=2$, the filter coincides with a third-order Nordsieck method (Nordsieck 1962) if the filter is in the steady state, i.e., after the sequence of error covariance matrices has converged. These results neither cover filters with the integrated Ornstein–Uhlenbeck process (IOUP) prior (Magnani et al. 2017) nor nonzero noise models on evaluations of *f*.

In this paper, we directly prove convergence rates without first fitting the filter to existing methods, and thereby lift many of the above restrictions on the convergence rates. While the more-recent work by Tronarp et al. (2020) also provide convergence rates of estimators of *x* in the Bayesian ODE filtering/smoothing paradigm, they concern the maximum a posteriori estimator (as computed by the iterated extended Kalman ODE smoother), and therefore differ from our convergence rates of the filtering mean (as computed by the Kalman ODE filter).

### Contribution

Our main results—Theorems 8 and 14—provide local and global convergence rates of the ODE filter when the step size *h* goes to zero. Theorem 8 shows local convergence rates of $h^{q+1}$ without the above-mentioned previous restrictions—i.e., for a generic Gaussian ODE filter for all $q \in {\mathbb {N}}$, both IBM and IOUP prior, flexible Gaussian initialization (see Assumptions 2 and 3), and arbitrary evaluation noise $R \ge 0$. As a first global convergence result, Theorem 14 establishes global convergence rates of $h^{q}$ in the case of $q=1$, the IBM prior and all fixed measurement uncertainty models *R* of order $p \in {[}1,\infty ]$ (see Assumption 4). This global rate of the worst-case error is matched by the contraction rate of the posterior credible intervals, as we show in Theorem 15. Moreover, we also give closed-form expressions for the steady states in the global case and illustrate our results as well as their possible generalizability to $q \ge 2$ by experiments in Sect. 9.

### Related work on probabilistic ODE solvers

The Gaussian ODE filter can be thought of as a self-consistent Bayesian decision agent that iteratively updates its prior belief ${\varvec{X}}$ over $x :[0,T] \rightarrow {\mathbb {R}}^d$ (and its first *q* derivatives) with information on ${\dot{x}}$ from evaluating *f*.^1^ For Gauss–Markov priors, it performs exact Bayesian inference and optimally (with respect to the $L^2$-loss) extrapolates along the time axis. Accordingly, all of its computations are deterministic and—due to its restriction to Gaussian distributions—only slightly more expensive than classical solvers. Experiments demonstrating competitive performance with classical methods are provided in Schober et al. (2019, Section 5).

Another line of work (comprising the methods from Chkrebtii et al. (2016); Conrad et al. (2017); Teymur et al. (2016); Lie et al. (2019); Abdulle and Garegnani (2020); Teymur et al. (2018)) introduces probability measures to ODE solvers in a fundamentally different way—by representing the distribution of all numerically possible trajectories with a set of sample paths. To compute these sample paths, Chkrebtii et al. (2016) draws them from a (Bayesian) Gaussian process (GP) regression; Conrad et al. (2017); Teymur et al. (2016); Lie et al. (2019); Teymur et al. (2018) perturb classical estimates after an integration step with a suitably scaled Gaussian noise; and Abdulle and Garegnani (2020) perturbs the classical estimate instead by choosing a stochastic step-size. While Conrad et al. (2017); Teymur et al. (2016); Lie et al. (2019); Abdulle and Garegnani (2020); Teymur et al. (2018) can be thought of as (non-Bayesian) ‘stochastic wrappers’ around classical solvers, which produce samples with the same convergence rate, Chkrebtii et al. (2016) employs—like the filter—GP regression to represent the belief on *x*. While the Gaussian ODE filter can convergence with polynomial order (see results in this paper), However, Chkrebtii et al. (2016) only show first-order convergence rates and also construct a sample representation of numerical errors, from which samples are drawn iteratively. A conceptual and experimental comparison between the filter and Chkrebtii et al. (2016) can be found in Schober et al. (2019). An additional numerical test against Conrad et al. (2017) was given by Kersting and Hennig (2016). Moreover, Tronarp et al. (2019) recently introduced a particle ODE filter, which combines a filtering-based solver with a sampling-based uncertainty quantification (UQ), and compared it numerically with Conrad et al. (2017) and Chkrebtii et al. (2016).

All of the above sampling-based methods can hence represent more expressive, non-Gaussian posteriors (as, e.g., desirable for bifurcations), but multiply the computational cost of the underlying method by the number of samples. ODE filters are, in contrast, not a perturbation of known methods, but novel methods designed for computational speed and for a robust treatment of intermediate uncertain values (such as the evaluations of *f* at estimated points). Unless parallelization of the samples in the sampling-based solvers is possible and inexpensive, one can spend the computational budget for generating additional samples on dividing the step size *h* by the number of samples, and can thereby polynomially decrease the error. Its Gaussian UQ, however, should not be regarded as the true UQ—in particular for chaotic systems whose uncertainty can be better represented by sampling-based solvers, see, e.g., Conrad et al. (2017, Figure 1) and Abdulle and Garegnani (2020, Figure 2)—but as a rough inexpensive probabilistic treatment of intermediate values and final errors which is supposed to, on average, guide the posterior mean towards the true *x*. Therefore, it is in a way more similar to classical non-stochastic solvers than to sampling-based stochastic solvers and, unlike sampling-based solvers, puts emphasis on computational speed over statistical accuracy. Nevertheless, its Gaussian UQ is sufficient to make the forward models in ODE inverse problems more ‘uncertainty-aware’; see Kersting et al. (2020, Section 3).

Accordingly, the convergence results in this paper concern the convergence rate of the posterior mean to the true solution, while the theoretical results from Teymur et al. (2016); Chkrebtii et al. (2016); Conrad et al. (2017); Lie et al. (2019); Abdulle and Garegnani (2020); Teymur et al. (2018) provide convergence rates of the variance of the non-Gaussian empirical measure of samples (and not for an individual sample).

### Relation to filtering theory

While Gaussian (Kalman) filtering was first applied to the solution of ODEs by Kersting and Hennig (2016) and Schober et al. (2019), it has previously been analyzed in the filtering, data assimilation as well as linear system theory community. The convergence results in this paper are concerned with its asymptotics when the step size *h* (aka time step between data points) goes to zero. In the classical filtering setting, where the data comes from an external sensor, this quantity is not treated as a variable, as it is considered a property of the data and not, like in our case, of the algorithm. Accordingly, the standard books lack such an analysis for $h \rightarrow 0$—see Jazwinski (1970); Anderson and Moore (1979); Maybeck (1979) for filtering, Law et al. (2015); Reich and Cotter (2015) for data assimilation and Callier and Desoer (1991) for linear system theory—and we believe that our convergence results are completely novel. It is conceivable that, also for these communities, this paper may be of interest in settings where the data collection mechanism can be actively chosen, e.g., when the frequency of the data can be varied or sensors of different frequencies can be used.

### Outline

The paper begins with a brief introduction to Gaussian ODE filtering in Sect. 2. Next, Sects. 3 and 5 provide auxiliary bounds on the flow map of the ODE and on intermediate quantities of the filter, respectively. With the help of these bounds, Sects. 6 and 7 establish local and global convergence rates of the filtering mean, respectively. In light of these rates, Sect. 8 analyses for which measurement noise models the posterior credible intervals are well calibrated. These theoretical results are experimentally confirmed and discussed in Sect. 9. Section 10 concludes with a high-level discussion.

### Notation

We will use the notation $[n] :=\{0,\dots ,n-1\}$. For vectors and matrices, we will use zero-based numbering, e.g., $x=(x_0,\dots ,x_{d-1}) \in {\mathbb {R}}^d .$ For a matrix $P \in {\mathbb {R}}^{n \times m}$ and $(i,j) \in [n] \times [m]$, we will write $P_{i,:} \in {\mathbb {R}}^{1 \times m}$ for the *i*th row and $P_{:,j}$ for the *j*th column of *P*. A fixed but arbitrary norm on ${\mathbb {R}}^d$ will be denoted by $\Vert \cdot \Vert$. The minimum and maximum of two real numbers *a* and *b* will be denoted by $a \wedge b$ and $a \vee b$, respectively. Vectors that span all *q* modeled derivatives will be denoted by bold symbols, such as ${\varvec{x}}$.

## Gaussian ODE filtering

This section defines how a Gaussian filter can solve the IVP Eq. (1). In the various subsections, we first explain the choice of prior on *x*, then describe how the algorithm computes a posterior output from this prior (by defining a numerical integrator $\varvec{\varPsi }$), and add explanations on the measurement noise of the derivative observations. To alternatively understand how this algorithm can be derived as an extension of generic Gaussian filtering in probabilistic state space models, see the concise presentation in (Kersting et al. 2020, Supplement A).

### Prior on ${\varvec{x}}$

In PN, it is common (Hennig et al. 2015, Section 3(a)) to put a prior measure on the unknown solution *x*. Often, for fast Bayesian inference by linear algebra (Rasmussen and Williams 2006, Chapter 2), this prior is Gaussian. To enable GP inference in linear time by Kalman filtering (Särkkä 2013, Chapter 4.3), we further restrict the prior to Markov processes. As discussed in Särkkä and Solin (2019, Chapter 12.4), a wide class of such Gauss–Markov processes can be captured by a law of the (strong) solution (Øksendal 2003, Chapter 5.3) of a linear SDE with Gaussian initial condition. Here—as we, by Eq. (1), have information on at least one derivative of *x*—the prior also includes the first $q \in {\mathbb {N}}$ derivatives. Therefore, for all $j \in [d]$, we define the vector of time derivatives by ${\varvec{X}}_j = \left( X_j^{(0)}, \dots , X_j^{(q)} \right) ^ \intercal$. We define ${\varvec{X}}_{j}$ as a $(q + 1)$-dimensional stochastic process via the SDE

2 $$\begin{aligned}&\mathrm {d}{\varvec{X}}_{j}{(t)} = \left( \mathrm {d}X_{j}^{(0)}{(t)} , \dots , \mathrm {d}X_{j}^{(q-1)}{(t)} , \mathrm {d}X_{j}^{(q)}{(t)} \right) ^{\intercal } \nonumber \\&\quad = \begin{pmatrix} 0 &{} 1 &{} 0 \dots &{} 0 \\ \vdots &{} \ddots &{} \ddots &{} 0 \\ \vdots &{} \ddots &{} 0 &{} 1 \\ c_0 &{} \dots &{} \dots &{} c_q \end{pmatrix} \begin{pmatrix} X_{j}^{(0)}{(t)} \\ \vdots \\ X_{j}^{(q-1)}{(t)} \\ X_{j}^{(q)}{(t)} \end{pmatrix} \, \mathrm {d}t + \begin{pmatrix} 0 \\ \vdots \\ 0 \\ \sigma _j \end{pmatrix} \, \mathrm {d}B_{j}{(t)}, \end{aligned}$$

driven by mutually independent one-dimensional Brownian motions $\{B_{j};\ j\in [d]\}$ (independent of ${\varvec{X}}(0)$) scaled by $\sigma _j > 0$, with initial condition ${\varvec{X}}_{j}(0) \sim {\mathcal {N}} ( m_j(0) , P_j(0) )$. We assume that $\left\{ X_{j}(0);\ j \in [d] \right\}$ are independent. In other words, we model the unknown *i*th derivative of the *j*th dimension of the solution *x* of the IVP Eq. (1), denoted by $x^{(i)}_j$, as a draw from a real-valued, one-dimensional GP $X^{(i)}_j$, for all $i \in [q+1]$ and $j \in [d]$, such that $X^{(q)}_j$ is defined by $(c_0,\dots ,c_q)$ as well as the Brownian motion scale $\sigma _j$ and $X^{(i-1)}_j$ is defined to be the integral of $X^{(i)}_j$. Note that, by the independence of the components of the *d*-dimensional Brownian motion, the components $\left\{ \left\{ {\varvec{X}}_{j}(t);\ 0 \le t \le T \right\} ;\ j \in [d] \right\}$ of $\left\{ {\varvec{X}}(t);\ 0\le t\le T \right\}$ are independent^2^. The (strong) solution of Eq. (2) is a Gauss–Markov process with mean $m_j :[0,T] \rightarrow {\mathbb {R}}^{q+1}$ and covariance matrix $P_j :[0,T] \rightarrow {\mathbb {R}}^{(q+1)\times (q+1)}$ given by

3 $$\begin{aligned} m_j(t)&= A(t) m_j(0), \end{aligned}$$

4 $$\begin{aligned} P_j(t)&= A(t)P_j(0) A(t)^{\intercal } + {Q(t)}, \end{aligned}$$

where the matrices $A(t),\ Q(t) \in {\mathbb {R}}^{(q+1)\times (q+1)}$ yielded by the SDE Eq. (2) are known in closed form Särkkä (2006, Theorem 2.9) (see Eq. (77)). The precise choice of the prior stochastic process ${\varvec{X}}$ depends on the choice of $(c_0,\dots ,c_q) \in {\mathbb {R}}^{q+1}$ in Eq. (2). While the below algorithm works for all choices of *c*, we restrict our attention to the case of

5 $$\begin{aligned} (c_0,\dots ,c_q) :=(0,\dots ,0,-\theta ), \qquad \text {for some} \quad \theta \ge 0, \end{aligned}$$

where the *q*-times integrated Brownian motion (IBM) and the *q*-times integrated Ornstein–Uhlenbeck process (IOUP) with drift parameter $\theta$ is the unique solution of Eq. (2), in the case of $\theta = 0$ and $\theta > 0$, respectively, (Karatzas and Shreve 1991, Chapter 5: Example 6.8). In this case, the matrices *A* and *Q* from Eqs. (3) and (4) are given by

6 $$\begin{aligned} {A(t)_{ij}}&= {\left\{ \begin{array}{ll} {\mathbb {I}}_{i\le j} \frac{t^{j-i}}{(j-i)!}, &{} \text{ if } j\ne q, \\ {\frac{t^{q-i}}{(q-i)!} - \theta \sum \nolimits _{k=q+1-i}^{\infty } \frac{(-\theta )^{k+i-q-1} t^k}{ k! }} , &{} \text{ if } j=q, \end{array}\right. } \end{aligned}$$

7 $$\begin{aligned} {Q(t)_{ij}}&= \sigma ^2 \frac{t^{2q+1-i-j}}{(2q+1-i-j)(q-i)!(q-j)!} \nonumber \\&\quad + \varTheta \left( t^{2q+2-i-j}\right) . \end{aligned}$$

(Derivations of Eqs. (6) and (7), as well as the precise form of *Q* without $\varTheta (t^{2q+2-i-j})$, are presented in Appendix A.) Hence, for all $i \in [q+1]$, the prediction of step size *h* of the *i*th derivative from any state $u\in {\mathbb {R}}^{q+1}$ is given by

8 $$\begin{aligned} \left[ A(t) u \right] _i =&\sum _{k=i}^q \frac{t^{k-i}}{(k-i)!} u_k \nonumber \\&- \theta \left[ \sum _{k=q+1-i}^{\infty } \frac{(-\theta )^{k+i-q-1}}{k!} t^k \right] u_q. \end{aligned}$$

### The algorithm

To avoid the introduction of additional indices, we will define the algorithm $\varvec{\varPsi }$ for $d=1$; for statements on the general case of $d\in {\mathbb {N}}$ we will use the same symbols from Eq. (10)–(15) as vectors over the whole dimension—see, e.g., Eq. (31) for a statement about a general $r \in {\mathbb {R}}^d$. By the independence of the dimensions of ${\varvec{X}}$, due to Eq. (2), extension to $d \in {\mathbb {N}}$ amounts to applying $\varvec{\varPsi }$ to every dimension independently (recall Footnote 2). Accordingly, we may in many of the below proofs w.l.o.g. assume $d=1$. Now, as previously spelled out in Kersting and Hennig (2016); Schober et al. (2019), Bayesian filtering of ${\varvec{X}}$—i.e., iteratively conditioning ${\varvec{X}}$ on the information on $X^{(1)}$ from evaluations of *f* at the mean of the current conditioned ${X}^{(0)}$—yields the following numerical method $\varvec{\varPsi }$. Let ${\varvec{m}}(t) = (m^{(0)}(t),\dots ,m^{(q)}(t))^{\intercal } \in {\mathbb {R}}^{q+1}$ be an arbitrary state at some point in time $t \in [0,T]$ (i.e., $m^{(i)}(t)$ is an estimate for $x^{(i)}(t)$), and let $P(t) \in {\mathbb {R}}^{(q+1) \times (q+1)}$ be the covariance matrix of $x^{(i)}(t)$. For $t \in [0,T]$, let the current estimate of ${\varvec{x}}(t)$ be a normal distribution ${\mathcal {N}}({\varvec{m}}(t),P(t))$, i.e., the mean ${\varvec{m}}(t) \in {\mathbb {R}}^{q+1}$ represents the best numerical estimate (given data $\{y(h),\dots ,y(t)\}$, see Eq. (12)) and the covariance matrix $P(t) \in {\mathbb {R}}^{(q+1) \times (q+1)}$ its uncertainty. For the time step $t \rightarrow t+h$ of size $h>0$, the ODE filter first computes the prediction step consisting of *predictive mean*

9 $$\begin{aligned} {{\varvec{m}}^-(t+h)}&{:=A(h) {\varvec{m}}(t) \ \in {\mathbb {R}}^{q+1}, } \end{aligned}$$

and *predictive covariance*

10 $$\begin{aligned} P^-(t+h)&:=A(h)P(t)A(h)^{\intercal } + Q{(h)} \ \in {\mathbb {R}}^{(q+1)\times (q+1)}, \end{aligned}$$

with *A* and *Q* generally defined by Eq. (77) and, in the considered particular case of Eq. (5), by Eqs. (6) and (7). In the subsequent step, the following quantities are computed first: the *Kalman gain*

11 $$\begin{aligned} \varvec{\beta }(t+h)&= (\beta ^{(0)}(t+h), \dots , \beta ^{(q)}(t+h))^{\intercal } \nonumber \\&:=\frac{P^-(t+h)_{:1}}{(P^-(t+h))_{11} + R(t+h)} \in {\mathbb {R}}^{(q+1) \times 1}, \end{aligned}$$

the *measurement/data on* ${\dot{x}}$

12 $$\begin{aligned} y(t+h)&:=f\left( m^{-,(0)}(t+h) \right) \ \in {\mathbb {R}}, \end{aligned}$$

and *innovation/residual*

13 $$\begin{aligned} r(t+h)&:=y(t+h) - m^{-,(1)}(t+h) \ \in {\mathbb {R}}. \end{aligned}$$

Here, *R* denotes the variance of *y* (the ‘measurement noise’) and captures the squared difference between the data $y(t+h) = f(m^-(t+h))$ that the algorithm actually receives and the idealized data ${\dot{x}}(t+h) = f(x(t+h))$ that it ‘should’ receive (see Sect. 2.3). Finally, the mean and the covariance matrix are conditioned on this data, which yields the *updated mean*

14 $$\begin{aligned} {\varvec{\varPsi }_{P(t),h}({\varvec{m}}(t))}&:={\varvec{m}}(t+h) \nonumber \\&= {\varvec{m}}^-(t+h) + \varvec{\beta }(t+h) r(t+h), \end{aligned}$$

and the *updated covariance*

15 $$\begin{aligned} P(t+h)&:=P^-(t+h) - \frac{P^-(t+h)_{:,1}P^-(t+h)_{1,:}}{P^-(t+h)_{11} + R(t+h)}. \end{aligned}$$

This concludes the step $t \rightarrow t+h$, with the Gaussian distribution ${\mathcal {N}}({\varvec{m}}(t+h),P(t+h))$ over ${\varvec{x}}(t+h)$. The algorithm is iterated by computing ${\varvec{m}}(t+2h) :=\varvec{\varPsi }_{P(t+h),h}({\varvec{m}}(t+h))$ as well as repeating Eq. (10) and (15), with $P(t+h)$ instead of *P*(*t*), to obtain $P(t+2h)$. In the following, to avoid notational clutter, the dependence of the above quantities on *t*, *h* and $\sigma$ will be omitted if their values are unambiguous. Parameter adaptation reminiscent of classical methods (e.g., for $\sigma$ s.t. the added variance per step coincide with standard error estimates) has been explored in Schober et al. (2019, Section 4).

This filter is essentially an iterative application of Bayes rule (see, e.g., Särkkä (2013, Chapter 4)) based on the prior ${\varvec{X}}$ on ${\varvec{x}}$ specified by Eq. (2) (entering the algorithm via *A* and *Q*) and the measurement model $y \sim {\mathcal {N}}({\dot{x}},R)$. Since the measurement model is a likelihood by another name and therefore forms a complete Bayesian model together with the prior ${\varvec{X}}$, it remains to detail the measurement model (recall Sect. 2.1 for the choice of prior). Concerning the data generation mechanism for *y* Eq. (12), we only consider the maximum-a-posteriori point estimate of ${\dot{x}}(t)$ given ${\mathcal {N}}(m^{-,(0)}(t),P_{00}^-(t))$; a discussion of more involved statistical models for *y* as well as an algorithm box for the Gaussian ODE filter can be found in Schober et al. (2019, Subsection 2.2). Next, for lack of such a discussion for *R*, we will examine different choices of *R*—which have proved central to the UQ of the filter (Kersting and Hennig 2016) and will turn out to affect global convergence properties in Sect. 7.

### Measurement noise *R*

Two sources of uncertainty add to *R*(*t*): noise from imprecise knowledge of *x*(*t*) and *f*. Given *f*, previous integration steps of the filter (as well as an imprecise initial value) inject uncertainty about how close $m^-(t)$ is to *x*(*t*) and how close $y = f(m^-(t))$ is to ${\dot{x}}(t)) = f(x(t))$. This uncertainty stems from the discretization error $\Vert m^{-,(0)}(t) - x(t) \Vert$ and, hence, tends to increase with *h*. Additionally, there can be uncertainty from a misspecified *f*, e.g., when *f* has estimated parameters, or from numerically imprecise evaluations of *f*, which can be added to *R*—a functionality which classical solvers do not possess. In this paper, since *R* depends on *h* via the numerical uncertainty on *x*(*t*), we analyze the influence of noise *R* of order $p \in [1,\infty ]$ (see Assumption 4) on the quality of the solution to illuminate for which orders of noise we can trust the solution to which extent and when we should, instead of decreasing *h*, rather spend computational budget on specifying or evaluating *f* more precisely. The explicit dependence of the noise on its order *p* in *h* resembles, despite the fundamentally different role of *R* compared to additive noise in Conrad et al. (2017); Abdulle and Garegnani (2020), the variable *p* in Conrad et al. (2017, Assumption 1) and Abdulle and Garegnani (2020, Assumption 2.2) in the sense that the analysis highlights how uncertainty of this order can still be modeled without breaking the convergence rates. (Adaptive noise models are computationally feasible (Kersting and Hennig 2016) but lie outside the scope of our analysis.)

## Regularity of flow

Before we proceed to the analysis of $\varvec{\varPsi }$, we provide all regularity results necessary for arbitrary $q,d \in {\mathbb {N}}$ in this section.

### Assumption 1

The vector field $f \in C^{q} ( {\mathbb {R}}^d; {\mathbb {R}}^d )$ is globally Lipschitz, and all its derivatives of order up to *q* are uniformly bounded and globally Lipschitz, i.e., there exists some $L>0$ such that $\Vert D^{\alpha } f \Vert _{\infty } \le L$ for all multi-indices $\alpha \in {\mathbb {N}}_0^{d}$ with $1 \le \sum _i \alpha _i \le q$, and $\Vert D^{\alpha } f(a) - D^{\alpha } f(b) \Vert \le L \Vert a - b \Vert$ for all multi-indices $\alpha \in {\mathbb {N}}_0^{d}$ with $0 \le \sum _i \alpha _i \le q$.

Assumption 1 and the Picard–Lindelöf theorem imply that the solution *x* is a well-defined element of $C^{q+1} ( [0,T];{\mathbb {R}}^d )$. For $i \in [q+1]$, we denote $\frac{\mathrm {d}^i x}{\mathrm {d}t^i}$ by $x^{(i)}$. Recall that, by a bold symbol, we denote the vector of these derivatives: ${\varvec{x}} \equiv ( x^{(0)}, \dots , x^{(q)} )^{\intercal }$. In particular, the solution *x* of Eq. (1) is denoted by $x^{(0)}$. Analogously, we denote the flow of the ODE Eq. (1) by $\varPhi ^{(0)}$, i.e., $\varPhi _t^{(0)}(x_0) \equiv x^{(0)}(t)$, and, for all $i\in [q+1]$, its *i*th partial derivative with respect to *t* by $\varPhi ^{(i)}$, so that $\varPhi _t^{(i)}(x_0) \equiv x^{(i)}(t)$.

### Lemma 1

Under Assumption 1, for all $a \in {\mathbb {R}}^d$ and all $h>0$,

16 $$\begin{aligned} \left\| \varPhi _h^{(i)}(a) - \sum _{k=i}^q \frac{h^{k-i}}{(k-i)!} \varPhi _0 ^{(k)}(a) \right\| \le Kh^{q+1-i}. \end{aligned}$$

Here, and in the sequel, $K>0$ denotes a constant independent of *h* and $\theta$ which may change from line to line.

### Proof

By Assumption 1, $\varPhi ^{(q+1)}$ exists and is bounded by $\Vert \varPhi ^{(q+1)} \Vert \le L$, which can be seen by applying the chain rule *q* times to both sides of Eq. (1). Now, applying $\Vert \varPhi ^{(q+1)} \Vert \le L$ to the term $\varPhi _{\tau }^{(q+1)}(a)$ (for some $\tau \in (0,h)$) in the Lagrange remainder of the $(q-i)$th-order Taylor expansion of $\varPhi _h^{(i)}(a)$ yields Eq. (16). $\square$

### Lemma 2

Under Assumption 1 and for all sufficiently small $h>0$,

17 $$\begin{aligned} \sup _{a\ne b \in {\mathbb {R}}^d} \frac{\left\| \varPhi ^{(0)}_h(a) - \varPhi ^{(0)}_h(b) \right\| }{\left\| a - b \right\| } \le 1+2Lh. \end{aligned}$$

### Proof

Immediate corollary of Teschl (2012, Theorem 2.8). $\square$

Global convergence (Sect. 7) will require the following generalization of Lemma 2.

### Lemma 3

Let $q=1$. Then, under Assumption 1 and for all sufficiently small $h>0$,

18 $$\begin{aligned} \sup _{a\ne b \in {\mathbb {R}}^d} \frac{{\left| \left| \left| \varvec{\varPhi }_h(a) - \varvec{\varPhi }_h(b) \right| \right| \right| }_h}{\left\| a - b \right\| } \le 1+Kh, \end{aligned}$$

where given the norm $\Vert \cdot \Vert$ on ${\mathbb {R}}^d$ and $h>0$, the new norm ${\left| \left| \left| \cdot \right| \right| \right| }_h$ on ${\mathbb {R}}^{(q+1)\times d}$ is defined by

19 $$\begin{aligned} {\left| \left| \left| a \right| \right| \right| }_h :=\sum _{i=0}^q h^i \left\| a_{i,:} \right\| . \end{aligned}$$

### Remark 1

The necessity of ${\left| \left| \left| \cdot \right| \right| \right| }_h$ stems from the fact that—unlike other ODE solvers—the ODE filter $\varvec{\varPsi }$ additionally estimates and uses the first *q* derivatives in its state ${\varvec{m}} \in {\mathbb {R}}^{(q+1)\times d}$, whose development cannot be bounded in $\Vert \cdot \Vert$, but in ${\left| \left| \left| \cdot \right| \right| \right| }_h$. The norm ${\left| \left| \left| \cdot \right| \right| \right| }_h$ is used to make rigorous the intuition that the estimates of the solution’s time derivative are ‘one order of *h* worse per derivative.’

### Proof

We bound the second summand of

20 $$\begin{aligned}&{\left| \left| \left| \varvec{\varPhi }_h(a) - \varvec{\varPhi }_h(b) \right| \right| \right| }_h \quad {\mathop {=}\limits ^{\text {eq. }(19)}} \nonumber \\&\quad \underbrace{\Big \Vert \varPhi ^{(0)}_h(a) - \varPhi ^{(0)}_h(b) \Big \Vert }_{\le (1+2Lh) \Vert a - b \Vert , \text { by }eq.\, (17)} + \ h \Big \Vert \underbrace{\varPhi ^{(1)}_h(a)}_{=f\left( \varPhi ^{(0)}_h(a) \right) } - \underbrace{\varPhi ^{(1)}_h(b)}_{=f\left( \varPhi ^{(0)}_h(b) \right) } \Big \Vert \end{aligned}$$

by

21 $$\begin{aligned} \left\| f\left( \varPhi ^{(0)}_h(a)\right) - f\left( \varPhi ^{(0)}_h(b) \right) \right\|&{\mathop {\le }\limits ^{\text {Ass. }1}} \\ L \left\| \varPhi ^{(0)}_h(a) - \varPhi ^{(0)}_h(b) \right\|&{\mathop {\le }\limits ^{\text {eq. }(17)}} L(1+2Lh) \left\| a - b \right\| . \nonumber \end{aligned}$$

Inserting Eq. (21) into Eq. (20) concludes the proof. $\square$

## The role of the state misalignments $\delta$

In Gaussian ODE filtering, the interconnection between the estimates of the ODE solution $x(t)=x^{(0)}(t)$ and its first *q* derivatives $\{x^{(1)}(t), \dots , x^{(q)}(t)\}$ is intricate. From a purely analytical point of view, every possible estimate *m*(*t*) of *x*(*t*) comes with a fixed set of derivatives, which are implied by the ODE, for the following reason: Clearly, by Eq. (1), the estimate $m^{(1)}(t)$ of $x^{(1)}(t)$ ought to be *f*(*m*(*t*)). More generally (for $i \in [q+1]$) the estimate $m^{(i)}(t)$ of $x^{(i)}(t)$ is determined by the ODE as well. To see this, let us first recursively define $f^{(i)} :{\mathbb {R}}^d \rightarrow {\mathbb {R}}^d$ by $f^{(0)}(a) :=a$, $f^{(1)}(a) :=f(a)$ and $f^{(i)}(a) :=[ \nabla _x f^{(i-1)} \cdot f ](a)$. Now, differentiating the ODE, Eq. (1), $(i-1)$-times by the chain rule yields

22 $$\begin{aligned} x^{(i)}(t) = f^{(i-1)}(t)\left( x^{(0)}(t)\right) , \end{aligned}$$

which implies that $m^{(i)}(t)$ ought to be $f^{(i-1)}(t)\left( m^{(0)}(t)\right)$ Since

23 $$\begin{aligned} \varPhi _0^{(i)} \left( m^{(0)}(nh) \right) = f^{(i-1)}\left( m^{(0)}(nh)\right) \end{aligned}$$

(which we prove in Appendix E), this amounts to requiring that

24 $$\begin{aligned} m^{(i)}(t) \overset{!}{=} \varPhi _0^{(i)} \left( m^{(0)}(nh) \right) . \end{aligned}$$

Since $\varPhi _0^{(i)}$ is (recall Sect. 3) the *i*th time derivative of the flow map $\varPhi ^{(0)}$ at $t=0$, this simply means that $m^{(i)}(t)$ would be set to the ‘true’ derivatives in the case where the initial condition of the ODE, Eq. (1), is $x(0) = m^{(0)}(t)$ instead of $x(0)=x_0$—or, more loosely speaking, that the derivative estimates $m^{(i)}(t)$ are forced to comply with $m^{(0)}(t)$, irrespective of our belief $x^{(i)}(t) \sim {\mathcal {N}}(m^{(i)}(t),P_{ii}(t))$. The Gaussian ODE filter, however, does not use this (intractable) analytical approach. Instead, it jointly models and infers $x^{(0)}(t)$ and its first *q* derivatives $\{x^{(1)}(t),\dots ,x^{(q)}(t) \}$ in a state space ${\varvec{X}}$, as detailed in Sect. 2. The thus-computed filtering mean estimates $m^{(i)}(t)$ depend not only on the ODE but also on the statistical model—namely on the prior (SDE) and measurement noise *R*; recall Sects. 2.1 and 2.3. In fact, the analytically desirable derivative estimate, Eq. (24), is, for $i=1$, only satisfied if $R=0$ (which can be seen from Eq. (14)), and generally does not hold for $i\ge 2$ since both $f^{(i-1)}$ and $\varPhi ^{(i)}$ are inaccessible to the algorithm. The numerical example in Appendix C clarifies that $\delta ^{(i)}$ is likely to be strictly positive, even after the first step $0 \rightarrow h$.

This inevitable mismatch, between exact analysis and approximate statistics, motivates the following definition of the $i{\mathrm{th}}$ state *i*th *state misalignment at time* *t*:

25 $$\begin{aligned} \delta ^{(i)}(t) :=\left\| m^{(i)}(t) - \varPhi _0^{(i)}\left( m^{(0)}(t)\right) \right\| \ge 0. \end{aligned}$$

Intuitively speaking, $\delta ^{(i)}(t)$ quantifies how large this mismatch is for the *i*th derivative at time *t*. Note that $\delta ^{(i)}(t) = 0$ if and only if Eq. (24) holds—i.e., for $i=1$ iff $R=0$ (which can be seen from Eq. (14)) and only by coincidence for $i\ge 2$ since both $f^{(i-1)}$ and $\varPhi ^{(i)}_0$ are inaccessible to the algorithm. (Since $\varPhi ^{(0)}_0 = {\text {Id}}$, $\delta ^{(0)}(t)=0$ for all *t*.)

The possibility of $\delta ^{(i)} > 0$, for $i\ge 1$, is inconvenient for the below worst-case analysis since (if Eq. (24) held true and $\delta ^{(i)} \equiv 0$) the prediction step of the drift-less IBM prediction ($\theta =0$) would coincide with a Taylor expansions of the flow map $\varPhi ^{(i)}_0$; see Eq. (8). But, because $\delta ^{(i)} \ne 0$ in general, we have to additionally bound the influence of $\delta \ge 0$ which complicates the below proofs further.

Fortunately, we can *locally* bound the import of $\delta ^{(i)}$ by the easy Lemma 7 and *globally* by the more complicated Lemma 11 (see Sect. 7.3). Intuitively, these bounds demonstrate that the order of the deviation from a Taylor expansion of the state ${\varvec{m}} = [m^{(0)},\dots ,m^{(q)}]$ due to $\delta$ is not smaller than the remainder of the Taylor expansion. This means, more loosely speaking, that the import of the $\delta ^{(i)}$ is swallowed by the Taylor remainder. This effect is locally captured by Lemma 4 and globally by Lemma 12. The global convergence rates of $\delta ^{(i)}(T)$, as provided by Lemma 12, are experimentally demonstrated in Appendix D.

## Auxiliary bounds on intermediate quantities

Recall from Eq. (5) that $\theta = 0$ and $\theta >0$ denote the cases of IBM and IOUP prior with drift coefficient $\theta$, respectively. The ODE filter $\varPsi$ iteratively computes the filtering mean ${\varvec{m}}(nh)=( m^{(0)}(nh),\dots , m^{(q)}(nh) )^{\intercal } \in {\mathbb {R}}^{(q+1)}$ as well as error covariance matrices $P(nh) \in {\mathbb {R}}$ on the mesh $\{ nh \}_{n=0}^{T/h}$. (Here and in the following, we assume w.l.o.g. that $T/h \in {\mathbb {N}}$.) Ideally, the truncation error over all derivatives

26 $$\begin{aligned} { \varvec{\varepsilon }(nh) :=(\varepsilon ^{(0)}(nh), \dots , \varepsilon ^{(q)}(nh) )^{\intercal } :={\varvec{m}}(nh) - {\varvec{x}}(nh), } \end{aligned}$$

falls quickly as $h \rightarrow 0$ and is estimated by the standard deviation $\sqrt{P_{00}(nh)}$. Next, we present a classical worst-case convergence analysis over all *f* satisfying Assumption 1; see Sect. 10 for a discussion of the desirability and feasibility of an average-case analysis. To this end, we bound the added error of every step by intermediate values, defined in Eqs. (11) and (13),

27 $$\begin{aligned} \varDelta ^{(i)}((n+1)h)&:=\left\| \varPsi _{P(nh),h}^{(i)}({\varvec{m}}(nh)) - \varPhi _{h}^{(i)} \left( m^{(0)}(nh) \right) \right\| \end{aligned}$$

28 $$\begin{aligned}&\quad {\mathop {\le }\limits ^{\text {eq. }(14)}} \underbrace{\left\| \left( A(h) {\varvec{m}}(nh) \right) _i - \varPhi _{h}^{(i)} \left( m^{(0)}(nh) \right) \right\| }_{=:\varDelta ^{-(i)}((n+1)h)} \nonumber \\&\qquad \qquad + \left\| \beta ^{(i)}({(n+1)h}) \right\| \left\| r({(n+1)h}) \right\| , \end{aligned}$$

and bound these quantities in the order $\varDelta ^{-(i)}$, *r*, $\beta ^{(i)}$. These bounds will be needed for the local and global convergence analysis in Sects. 6 and 7, respectively. Note that, intuitively, $\varDelta ^{-(i)}((n+1)h)$ and $\varDelta ^{(i)}((n+1)h)$ denote the *additional* numerical error which is added in the $(n+1)$th step to the *i*th derivative of the predictive mean $m^{-,(i)}(t+h)$ and the updated mean $m^{(i)}(t+h)$, respectively.

### Lemma 4

Under Assumption 1, for all $i \in [q+1]$ and all $h>0$,

29 $$\begin{aligned} \varDelta ^{-(i)}((n+1)h) \le&K \left[ 1 + {\theta \left\| m^{(q)}(nh) \right\| } \right] h^{q+1-i} \nonumber \\&+ \sum _{k=i}^q \frac{h^{k-i}}{(k-i)!}\delta ^{(k)}(nh). \end{aligned}$$

### Proof

We may assume, as explained in Sect. 2.2, without loss of generality that $d=1$. We apply the triangle inequality to the definition of $\varDelta ^{-(i)}((n+1)h)$, as defined in Eq. (28), which, by Eq. (8), yields

30 $$\begin{aligned}&\varDelta ^{-(i)}((n+1)h) \quad \nonumber \\&\quad \le \sum _{k=i}^q \frac{h^{k-i}}{(k-i)!} \delta ^{(k)}(nh) + {K \theta \left| m^{(q)}(nh) \right| h^{q+1-i}} \nonumber \\&\qquad + \underbrace{\left| \sum _{l=i}^q \frac{h^{l-i}}{(l-i)!} \varPhi _{0}^{(l)}\left( m^{(0)}(nh) \right) - \varPhi _h^{(i)}\left( m^{(0)}(nh) \right) \right| }_{\le Kh^{q+1-i}, \text { by eq. } (16)}. \end{aligned}$$

$$\square $$

### Lemma 5

Under Assumption 1 and for all sufficiently small $h>0$,

31 $$\begin{aligned} \left\| r((n+1)h) \right\| \le&K \left[ 1 + {\theta \left\| m^{(q)}(nh) \right\| } \right] h^q \nonumber \\&+ K \sum _{k=1}^q \frac{h^{k-1}}{(k-1)!} \delta ^{(k)}(nh). \end{aligned}$$

### Proof

See Appendix F. $$\square $$

To bound the Kalman gains $\varvec{\beta }(nh)$, we first need to assume that the orders of the initial covariance matrices are sufficiently high (matching the latter required orders of the initialization error; see Assumption 3).

### Assumption 2

The entries of the initial covariance matrix *P*(0) satisfy, for all $k,l \in [q+1]$, $\Vert P(0)_{k,l} \Vert \le K_0 h^{2q+1-k-l}$, where $K_0>0$ is a constant independent of *h*.

We make this assumption, as well as Assumption 3, explicit (instead of just making the stronger assumption of exact initializations with zero variance), because it highlights how statistical or numerical uncertainty on the initial value effects the accuracy of the output of the filter—a novel functionality of PN with the potential to facilitate a management of the computational budget across a computational chain with respect to the respective perturbations from different sources of uncertainty (Hennig et al. 2015, Section 3(d)).

### Lemma 6

Under Assumption 2, for all $i\in [q+1]$ and for all $h>0$, $\Vert \beta ^{(i)}(h) \Vert \le K h^{1-i}$.

### Proof

Again, w.l.o.g. $d=1$. Application of the orders of *A* and *Q* from Eqs. (6) and (7), the triangle inequality and Assumption 2 to the definition of $P^-$ in Eq. (10) yields

32 $$\begin{aligned} \left| P^-(h)_{k,l} \right|&{\mathop {\le }\limits ^{\text {eq. }(10)}} \left| \left[ A(h)P(0)A(h)^{\intercal } \right] _{k,l} \right| + \left| Q{(h)}_{k,l} \right| \nonumber \\&{\mathop {\le }\limits ^{\text {eqs. }(6),(7)}} K \Bigg [ \sum _{a=k}^{q} \sum _{b=l}^q \left| P(0)_{a,b} \right| h^{a+b-k-l} \nonumber \\&\qquad \qquad + 2\theta \sum _{b=l}^{q-1} \left| P(0)_{q,b} \right| \nonumber \\&\qquad \qquad + \theta ^2 \left| P(0)_{q,q} \right| + h^{2q+1-k-l} \Bigg ] \nonumber \\&{\mathop {\le }\limits ^{\text {Ass. }2}} K {[1 + \theta + \theta ^2 ]} h^{2q+1-k-l}. \end{aligned}$$

Recall that *P* and *Q* are (positive semi-definite) covariance matrices; hence, $P^-(h)_{1,1} \ge Kh^{2q-1}$. Inserting these orders into the definition of $\beta ^{(i)}$ (Eq. (11)), recalling that $R \ge 0$, and removing the dependence on $\theta$ by reducing the fraction conclude the proof. $\square$

## Local convergence rates

With the above bounds on intermediate algorithmic quantities (involving state misalignments $\delta ^{(i)}$) in place, we only need an additional assumption to proceed—via a bound on $\delta ^{(i)}(0)$—to our first main result on local convergence orders of $\varvec{\varPsi }$.

### Assumption 3

The initial errors on the initial estimate of the *i*th derivative $m^{(i)}(0)$ satisfy $\Vert \varepsilon ^{(i)}(0) \Vert = \Vert m^{(i)}(0) - x^{(i)}(0) \Vert \le K_0 h^{q+1-i}$. (This assumption is, like Assumption 2, weaker than the standard assumption of exact initializations.)

### Lemma 7

Under Assumptions 1 and 3, for all $i\in {[}q+1]$ and for all $h>0$, $\delta ^{(i)}(0) \le Kh^{q+1-i}.$

### Proof

The claim follows, using Assumptions 1 and 3, from

33 $$\begin{aligned} \delta ^{(i)}(0) \le&\underbrace{\left\| m^{(i)}(0) - x^{(i)}(0) \right\| }_{= \Vert \varepsilon ^{(i)}(0) \Vert \le K_0 h^{q+1-i}} \nonumber \\&+ \underbrace{\left\| f^{(i-1)} \left( x^{(0)}(0) \right) - f^{(i-1)} \left( m^{(0)}(0) \right) \right\| }_{\le L \Vert \varepsilon ^{(0)}(0) \Vert \le L K_0 h^{q+1}}. \end{aligned}$$

$$\square $$

Now, we can bound the local truncation error $\varepsilon ^{(0)}(h)$ as defined in Eq. (26).

### Theorem 8

(Local Truncation Error) Under the Assumptions 1 to 3 and for all sufficiently small $h>0$,

34 $$\begin{aligned} \left\| \varepsilon ^{(0)}(h) \right\| \le {\left| \left| \left| \varvec{\varepsilon } (h) \right| \right| \right| }_h \le K \left[ 1 + {\theta \left\| m^{(q)}(0) \right\| } \right] h^{q+1}. \end{aligned}$$

### Proof

By the triangle inequality for ${\left| \left| \left| \cdot \right| \right| \right| }_h$ and subsequent application of Lemma 3 and Assumption 3 to the second summand of the resulting inequality, we obtain

35 $$\begin{aligned} {\left| \left| \left| \varvec{\varepsilon }(h) \right| \right| \right| }_h \le&\underbrace{{\left| \left| \left| \varvec{\varPsi }_{P(0),h} \left( {\varvec{m}}(0) \right) - \varvec{\varPhi }_h \left( x^{(0)}(0) \right) \right| \right| \right| }_h}_{= \sum _{i=0}^q h^i \varDelta ^{(i)}(h), \text { by eq. }(27)} \nonumber \\&+ \underbrace{{\left| \left| \left| \varvec{\varPhi }_h \left( x^{(0)}(0) \right) - \varvec{\varPhi }_h \left( m^{(0)}(0) \right) \right| \right| \right| }_h}_{\le (1+Kh) \Vert \varepsilon ^{(0)}(0) \Vert \le Kh^{q+1} } . \end{aligned}$$

The remaining bound on $\varDelta ^{(i)}(h)$, for all $i \in [q+1]$ and sufficiently small $h>0$, is obtained by insertion of the bounds from Lemmas 4 to 6 (in the case of $n=0$), into Eq. (28):

36 $$\begin{aligned} \varDelta ^{(i)}(h)&\le K \left[ 1 + {\theta \left\| m^{(q)}(0) \right\| } \right] h^{q+1-i} \nonumber \\&\qquad \quad + {K \sum _{k=1}^q \frac{h^{k-1}}{(k-1)!} \delta ^{(k)}(nh)} \end{aligned}$$

37 $$\begin{aligned}&{\mathop {\le }\limits ^{\text {Lemma } 7}} K \left[ 1 + {\theta \left\| m^{(q)}(0) \right\| } \right] h^{q+1-i} . \end{aligned}$$

Insertion of Eq. (37) into Eq. (35) and $\Vert \varepsilon ^{(0)}(h) \Vert \le {\left| \left| \left| \varvec{\varepsilon }(h) \right| \right| \right| }_h$ (by Eq. (19)) concludes the proof. $\square$

### Remark 2

Theorem 8 establishes a bound of order $h^{q+1}$ on the local truncation error $\varepsilon ^{(0)}(h)$ on *x*(*h*) after one step *h*.

Moreover, by the definition Eq. (19) of ${\left| \left| \left| \cdot \right| \right| \right| }_h$, this theorem also implies additional bounds of order $h^{q+1-i}$ on the error $\varepsilon ^{(i)}(h)$ on the *i*th derivative $x^{(i)}(h)$ for all $i \in [q+1]$. Such derivative bounds are (to the best of our knowledge) not available for classical numerical solvers, since they do not explicitly model the derivatives in the first place. These bounds could be useful for subsequent computations based on the ODE trajectory (Hennig et al. 2015).

Unsurprisingly, as the mean prediction (recall Eq. (8)) deviates from a pure *q*th order Taylor expansion by $K\theta \Vert m^{(q)}(0) \Vert h^{q+1}$ for an IOUP prior (i.e., $\theta > 0$ in Eq. (5)), the constant in front of the local $h^{q+1}$ convergence rate depends on both $\theta$ and $m^{(q)}(0)$ in the IOUP case. A global analysis for IOUP is therefore more complicated than for IBM: Recall from Eq. (8) that, for $q=1$, the mean prediction for $x((n+1)h)$ is

38 $$\begin{aligned}&\begin{pmatrix} m^{-,(0)}((n+1)h) \\ m^{-,(1)}((n+1)h) \end{pmatrix} \quad {\mathop {=}\limits ^{\text {eq. }(8)}} \nonumber \\&\begin{pmatrix} m^{(0)}(nh) + h m^{(1)}(nh) - \theta \left[ \frac{h^2}{2!} + {\mathcal {O}}(h^3) \right] m^{(1)}(nh) \\ e^{-\theta h} m^{-,(1)}(nh) \end{pmatrix}, \end{aligned}$$

which pulls both $m^{-,(0)}$ and $m^{-,(1)}$ towards zero (or some other prior mean) compared to the prediction given by its Taylor expansion for $\theta = 0$. While this is useful for ODEs converging to zero, such as $\dot{x} = -x$, it is problematic for diverging ODEs, such as $\dot{x} = x$ (Magnani et al. 2017). As shown in Theorem 8, this effect is asymptotically negligible for local convergence, but it might matter globally and, therefore, might necessitate stronger assumptions on *f* than Assumption 1, such as a bound on $\Vert f \Vert _{\infty }$ which would globally bound $\{ y(nh);\ n=0,\dots ,T/h \}$ and thereby $\{m^{(1)}(nh);\ n=0,\dots ,T/h\}$ in Eq. (38). It is furthermore conceivable that a global bound for IOUP would depend on the relation between $\theta$ and $\Vert f \Vert _{\infty }$ in a non-trivial way. The inclusion of IOUP ($\theta > 0$) would hence complicate the below proofs further. Therefore, we restrict the following first global analysis to IBM ($\theta = 0$).

## Global analysis

As explained in Remark 2, we only consider the case of the IBM prior, i.e., $\theta = 0$, in this section. Moreover, we restrict our analysis to $q=1$ in this first global analysis. Although we only have definite knowledge for $q=1$, we believe that the convergence rates might also hold for higher $q \in {\mathbb {N}}$—which we experimentally test in Sect. 9.1. Moreover, we believe that proofs analogous to the below proofs might work out for higher $q \in {\mathbb {N}}$ and that deriving a generalized version of Proposition 10 for higher *q* is the bottleneck for such proofs (see Sect. 10 for a discussion of these restrictions).

While, for local convergence, all noise models *R* yield the same convergence rates in Theorem 8, it is unclear how the order of *R* in *h* (as described in Sect. 2.3) affects global convergence rates: e.g., for the limiting case $R \equiv Kh^0$, the steady-state Kalman gains $\varvec{\beta }^{\infty }$ would converge to zero (see Eqs. (43) and 44 below) for $h \rightarrow 0$, and hence the evaluation of *f* would not be taken into account—yielding a filter $\varvec{\varPsi }$ which assumes that the evaluations of *f* are equally off, regardless of $h>0$, and eventually just extrapolates along the prior without global convergence of the posterior mean ${\varvec{m}}$. For the opposite limiting case $R\equiv \lim _{p \rightarrow \infty } K h^p \equiv 0$, it has already been shown in Schober et al. (2019, Proposition 1 and Theorem 1) that—in the steady state and for $q=1,2$—the filter $\varvec{\varPsi }$ inherits global convergence rates from known multistep methods in Nordsieck form Nordsieck (1962). To explore a more general noise model, we assume a fixed noise model $R \equiv K h^p$ with arbitrary order *p*.

In the following, we analyze how small *p* can be in order for $\varvec{\varPsi }$ to exhibit fast global convergence (cf. the similar role of the order *p* of perturbations in Conrad et al. (2017, Assumption 1) and Abdulle and Garegnani (2020, Assumption 2.2)). In light of Theorem 8, the highest possible global convergence rate is ${\mathcal {O}}(h)$—which will indeed be obtained for all $p \in [1,\infty ]$ in Theorem 14. Since every extrapolation step of $\varvec{\varPsi }$ from *t* to $t+h$ depends not only on the current state, but also on the covariance matrix *P*(*t*)—which itself depends on all previous steps—$\varPsi$ is neither a single-step nor a multistep method. Contrary to Schober et al. (2019), we do not restrict our theoretical analysis to the steady-state case, but provide our results under the weaker Assumptions 2 and 3 that were already sufficient for local convergence in Theorem 8—which is made possible by the bounds Eqs. (48) and (49) in Proposition 10.

### Outline of global convergence proof

The goal of the following sequence of proofs in Sect. 7 is Theorem 14. It is proved by a special version of the discrete Grönwall inequality (Clark 1987) whose prerequisite is provided in Lemma 13. This Lemma 13 follows from Lemma 3 (on the regularity of the flow map $\varvec{\varPhi }_t$) as well as Lemma 12 which provides a bound on the maximal increment of the numerical error stemming from local truncation errors. For the proof of Lemma 12, we first have to establish

(i) global bounds on the Kalman gains $\beta ^{(0)}$ and $\beta ^{(1)}$ by the inequalities Eqs. (48) and (49) in Proposition 10, and
(ii) a global bound on the state misalignment $\delta ^{(1)}$ in Lemma 11.

In Sects. 7.2–7.4, we will collect these inequalities in the order of their numbering to subsequently prove global convergence in Sect. 7.5.

### Global bounds on Kalman gains

Since we will analyze the sequence of covariance matrices and Kalman gains using contractions in Proposition 10, we first introduce the following generalization of Banach fixed-point theorem (BFT).

#### Lemma 9

Let $({\mathcal {X}},d)$ be a non-empty complete metric space, $T_n :{\mathcal {X}} \rightarrow {\mathcal {X}}$, $n\in {\mathbb {N}}$, a sequence of $L_n$-Lipschitz continuous contractions with $\sup _n L_n \le {{\bar{L}}} < 1$. Let $u_n$ be the fixed point of $T_n$, as given by BFT, and let $\lim _{n \rightarrow \infty } u_n = u^{*} \in {\mathcal {X}}$. Then, for all $x_0 \in {\mathcal {X}}$, the recursive sequence $x_n :=T_n(x_{n-1})$ converges to $u^{*}$ as $n \rightarrow \infty$.

#### Proof

See Appendix G. $$\square $$

In the following, we will assume that *T* is a multiple of *h*.

#### Proposition 10

For constant $R \equiv K h^p$ with $p \in [0,\infty ]$, the unique (attractive) steady states for the following quantities are

39 $$\begin{aligned} P_{11}^{-,\infty }&:=\lim _{n \rightarrow \infty } P_{11}^-(nh) \nonumber \\&= \frac{1}{2} \left( \sigma ^2 h + \sqrt{4\sigma ^2 R h + \sigma ^4 h^2} \right) , \end{aligned}$$

40 $$\begin{aligned} P_{11}^{\infty }&:=\lim _{n \rightarrow \infty } {P_{11}}(nh) \nonumber \\&= \frac{\left( \sigma ^2 h + \sqrt{4\sigma ^2 R h + \sigma ^4 h^2} \right) R}{\sigma ^2 h + \sqrt{4\sigma ^2 R h + \sigma ^4 h^2} + 2R}, \end{aligned}$$

41 $$\begin{aligned} P_{01}^{-,\infty }&:=\lim _{n \rightarrow \infty } P_{01}^{-}(nh) \nonumber \\&= \frac{\sigma ^4 h^2 + (2R + \sigma ^2 h) \sqrt{4\sigma ^2 Rh + \sigma ^4 h^2} + 4R\sigma ^2 h }{2(\sigma ^2 h + \sqrt{4\sigma ^2 R h + \sigma ^4 h^2})} h, \end{aligned}$$

42 $$\begin{aligned} P_{01}^{\infty }&:=\lim _{n \rightarrow \infty } P_{01}(nh) \nonumber \\&= \frac{R\sqrt{4R\sigma ^2 h + \sigma ^4 h^2} }{\sigma ^2 h + \sqrt{4\sigma ^2 R h + \sigma ^4 h^2}} h, \end{aligned}$$

43 $$\begin{aligned} \beta ^{\infty ,(0)}&:=\lim _{n \rightarrow \infty } \beta ^{(0)}(nh) \nonumber \\&= \frac{\sqrt{4R\sigma ^2 h + \sigma ^4 h^2} }{\sigma ^2 h + \sqrt{4\sigma ^2 R h + \sigma ^4 h^2}} h, \qquad \text {and} \end{aligned}$$

44 $$\begin{aligned} \beta ^{\infty ,(1)}&:=\lim _{n \rightarrow \infty } \beta ^{(1)}(nh) \nonumber \\&= \frac{\sigma ^2 h + \sqrt{4\sigma ^2 R h + \sigma ^4 h^2}}{ \sigma ^2 h + \sqrt{4\sigma ^2 R h + \sigma ^4 h^2} + 2R }. \end{aligned}$$

If furthermore Assumption 2 holds, then, for all sufficiently small $h>0$,

45 $$\begin{aligned} \max _{n \in [T/h + 1]} P_{11}^{-}(nh)&\le Kh^{1 \wedge \frac{p+1}{2}}, \end{aligned}$$

46 $$\begin{aligned} \max _{n \in [T/h + 1]} P_{11}(nh)&\le Kh^{p \vee \frac{p+1}{2} }, \end{aligned}$$

47 $$\begin{aligned} \max _{n \in [T/h + 1]} \left\| P_{01}(nh) \right\|&\le Kh^{p+1}, \end{aligned}$$

48 $$\begin{aligned} \max _{n \in [T/h + 1]} \left\| \beta ^{(0)}(nh) \right\|&\le Kh, \qquad \text {and} \end{aligned}$$

49 $$\begin{aligned} \max _{n \in [T/h + 1]} \left\| 1 - \beta ^{(1)}(nh) \right\|&\le Kh^{(p-1) \vee 0}. \end{aligned}$$

All of these bounds are sharp in the sense that they fail for any higher order in the exponent of *h*.

#### Remark 3

The recursions for *P*(*nh*) and $P^-(nh)$ given by Eqs. (10) and (15) follow a discrete algebraic Riccati equation (DARE)—a topic studied in many related settings (Lancaster and Rodman 1995). While the asymptotic behavior Eq. (39) of the completely detectable state $X^{(1)}$ can also be obtained using classical filtering theory (Anderson and Moore 1979, Chapter 4.4), the remaining statements of Proposition 10 also concern the undetectable state $X^{(0)}$ and are, to the best of our knowledge, not directly obtainable from existing theory on DAREs or filtering (which makes the following proof necessary). Note that, in the special case of no measurement noise ($R\equiv 0$), Eqs. (43) and (44) yield the equivalence of the filter in the steady state with the P(EC)1 implementation of the trapezoidal rule, which was previously shown in Schober et al. (2019, Proposition 1). For future research, it would be interesting to examine whether insertion of positive choices of *R* into Eqs. (43) and (44) can reproduce known methods as well.

#### Proof

See Appendix H. $$\square $$

### Global bounds on state misalignments

For the following estimates, we restrict the choice of *p* to be larger than $q=1$.

#### Assumption 4

The noise model is chosen to be $R\equiv Kh^p$, for $p \in {[}q,\infty ] = [1,\infty ]$, where $Kh^{\infty } : = 0$.

Before bounding the added deviation of $\varvec{\varPsi }$ from the flow $\varvec{\varPhi }$ per step, a global bound on the state misalignments defined in Eq. (25) is necessary. The result of the following lemma is discussed in Appendix D.

#### Lemma 11

Under Assumptions 1 to 4 and for all sufficiently small $h>0$,

50 $$\begin{aligned} \max _{n \in [T/h + 1]} \delta ^{(1)}(nh) \le {Kh}. \end{aligned}$$

#### Proof

See Appendix I. $$\square $$

See Lemma 11 for a experimental demonstration of Eq. (33).

### Prerequisite for discrete Grönwall inequality

Equipped with the above bounds, we can now prove a bound on the maximal increment of the numerical error stemming from local truncation errors which is needed to prove Eq. (56), the prerequisite for the discrete Grönwall inequality.

#### Lemma 12

Under Assumptions 1 to 4 and for all sufficiently small $h>0$,

51 $$\begin{aligned} \max _{n \in [T/h + 1]}&{\left| \left| \left| \varvec{\varPsi }_{P(nh),h}\left( {\varvec{m}}(nh) \right) - \varvec{\varPhi }_h \left( m^{(0)}(nh) \right) \right| \right| \right| }_h \nonumber \\&\quad \le {Kh^2}. \end{aligned}$$

#### Proof

By Eq. (19), we have

52 $$\begin{aligned}&{\left| \left| \left| \varvec{\varPsi }_{P(nh),h}\left( {\varvec{m}}(nh) \right) - \varvec{\varPhi }_h \left( m^{(0)}(nh) \right) \right| \right| \right| }_h \nonumber \\&\quad = S_1(h) + h S_2(h), \end{aligned}$$

with $S_1(h)$ and $S_2(h)$ defined and bounded by

53 $$\begin{aligned} S_1(h)&:=\left\| \varPsi ^{(0)}_h \left( {\varvec{m}}(nh) \right) - \varPhi _h^{(0)} \left( m^{(0)}(nh) \right) \right\| \nonumber \\&{\mathop {\le }\limits ^{\text {eq.}(28)}} \underbrace{\varDelta ^{-(0)} ((n+1)h)}_{{\mathop {\le }\limits ^{\text {eq. }(29)}} Kh^2 + \delta ^{(0)}(nh) + h \delta ^{(1)}(nh) } \nonumber \\&\qquad \quad + \underbrace{\left\| \beta ^{(0)}((n+1)h) \right\| }_{ {\mathop {\le }\limits ^{\text {eq. }(48)}} Kh} \underbrace{\left\| r((n+1)h) \right\| }_{{\mathop {\le }\limits ^{\text {eq. }(31)}} Kh + (1+Kh)\delta ^{(1)}(nh)} , \end{aligned}$$

and, analogously,

54 $$\begin{aligned} S_2(h)&:=\left\| \varPsi ^{(1)}_h \left( {\varvec{m}}(nh) \right) - \varPhi _h^{(1)} \left( m^{(0)}(nh) \right) \right\| \nonumber \\&{\mathop {\le }\limits ^{\text {eq. }(28)}} {\underbrace{\varDelta ^{-(1)}((n+1)h)}_{{\mathop {\le }\limits ^{\text {eq. } (29)}} Kh + \delta ^{(1)}(nh)} } \nonumber \\&\qquad \quad + \underbrace{\left\| \beta ^{(1)}((n+1)h) \right\| }_{{\mathop {\le }\limits ^{\text {eq. }(11)}} 1 } {\underbrace{\left\| r((n+1)h) \right\| }_{ {\mathop {\le }\limits ^{\text {eq. }(31)}} Kh + (1+Kh)\delta ^{(1)}(nh)}} \end{aligned}$$

Insertion of Eqs. (53) and (54) into Eq. (52) yields

55 $$\begin{aligned}&{\left| \left| \left| \varvec{\varPsi }_{P(nh),h}\left( {\varvec{m}}(nh) \right) - \varvec{\varPhi }_h \left( m^{(0)}(nh) \right) \right| \right| \right| }_h \nonumber \\&\quad \le Kh^2 + \delta ^{(0)}(nh) + Kh \delta ^{(1)}(nh) , \end{aligned}$$

which—after recalling $\delta ^{(0)}(nh) = 0$ and applying Lemma 11 to $\delta ^{(1)}(nh)$—implies Eq. (51). $\square$

The previous lemma now implies a suitable prerequisite for a discrete Grönwall inequality.

#### Lemma 13

Under Assumptions 1 to 4 and for all sufficiently small $h>0$,

56 $$\begin{aligned} {\left| \left| \left| \varvec{\varepsilon }\left( (n+1)h \right) \right| \right| \right| }_h \le {Kh^{2} + (1+Kh) \left\| \varepsilon ^{(0)}(nh) \right\| }. \end{aligned}$$

#### Proof

We observe, by the triangle inequality for the norm ${\left| \left| \left| \cdot \right| \right| \right| }_h$, that

57 $$\begin{aligned}&{\left| \left| \left| \varvec{\varepsilon } \left( (n+1)h \right) \right| \right| \right| }_h \nonumber \\&\quad = {\left| \left| \left| \varvec{\varPsi }_{P(nh),h} ({\varvec{m}}(nh)) - \varvec{\varPhi }_h \left( x^{(0)}(nh) \right) \right| \right| \right| }_h \nonumber \\&\quad \le {\left| \left| \left| \varvec{\varPsi }_{P(nh),h} ({\varvec{m}}(nh)) - \varvec{\varPhi }_{h} \left( m^{(0)}(nh) \right) \right| \right| \right| }_h \nonumber \\&\qquad + {\left| \left| \left| \varvec{\varPhi }_{h} \left( m^{(0)}(nh) \right) - \varvec{\varPhi }_{h} \left( x^{(0)}(nh) \right) \right| \right| \right| }_h. \end{aligned}$$

The proof is concluded by applying Lemma 12 to the first and Lemma 3 to the second summand of this bound (as well as recalling from Eq. (26) that $\Vert \varepsilon ^{(0)}(nh) \Vert = \Vert m^{(0)}(nh) - x^{(0)}(nh) \Vert$). $\square$

### Global convergence rates

With the above bounds in place, we can now prove global convergence rates.

#### Theorem 14

(Global truncation error) Under Assumptions 1 to 4 and for all sufficiently small $h>0$,

58 $$\begin{aligned} \max _{n \in [T/h + 1]} \left\| \varepsilon ^{(0)}(nh) \right\| \le \max _{n\in [T/h + 1]} {\left| \left| \left| \varvec{\varepsilon }(nh) \right| \right| \right| }_h \le {K(T)h}, \end{aligned}$$

where $K(T) > 0$ is a constant that depends on *T*, but not on *h*.

#### Remark 4

Theorem 14 not only implies that the truncation error $\Vert \varepsilon ^{(0)}(nh) \Vert$ on the solution of Eq. (1) has global order *h*, but also (by Eq. (19)) that the truncation error $\Vert \varepsilon ^{(1)}(nh) \Vert$ on the derivative is uniformly bounded by a constant *K* independent of *h*. The convergence rate of this theorem is sharp in the sense that it cannot be improved over all *f* satisfying Assumption 1 since it is one order worse than the local convergence rate implied by Theorem 8.

#### Proof

Using $\left\| \varepsilon ^{(0)}(nh) \right\| \le {\left| \left| \left| \varvec{\varepsilon }(nh) \right| \right| \right| }_h$ (due to Eq. (19)), the bound Eq. (56), a telescoping sum, and ${\left| \left| \left| \varvec{\varepsilon }(0) \right| \right| \right| }_h \le Kh^2$ (by Assumption 3), we obtain, for all sufficiently small $h>0$, that

59 $$\begin{aligned}&{\left| \left| \left| \varvec{\varepsilon } ((n+1)h) \right| \right| \right| }_h - {\left| \left| \left| \varvec{\varepsilon }(nh) \right| \right| \right| }_h \nonumber \\&\quad {\mathop {\le }\limits ^{\text {eq. }(19)}} {\left| \left| \left| \varvec{\varepsilon } ((n+1)h) \right| \right| \right| }_h - \left\| \varepsilon ^{(0)}(nh) \right\| \nonumber \\&\quad {\mathop {\le }\limits ^{\text {eq. }(56)}} { Kh^{2} + Kh \left\| \varepsilon ^{(0)}(nh) \right\| } \nonumber \\&\quad {\mathop {\le }\limits ^{\text {eq. }(19)}} {Kh^{2} + Kh {\left| \left| \left| \varvec{\varepsilon }(nh) \right| \right| \right| }_h} \nonumber \\&\quad {\mathop {=}\limits ^{\text {(tel. sum)}}} {Kh^{2} + {\left| \left| \left| \varvec{\varepsilon } (0) \right| \right| \right| }_h } \nonumber \\&\qquad \qquad \qquad + Kh \sum _{l=0}^{n-1} \left( {\left| \left| \left| \varvec{\varepsilon } ((l+1)h) \right| \right| \right| }_h - {\left| \left| \left| \varvec{\varepsilon }(lh) \right| \right| \right| }_h \right) \nonumber \\&\quad {\mathop {\le }\limits ^{({\left| \left| \left| \varvec{\varepsilon }(0) \right| \right| \right| }_h \le Kh^2)}} Kh^{2} \nonumber \\&\qquad \qquad \qquad + Kh \sum _{l=0}^{n-1} \left( {\left| \left| \left| \varvec{\varepsilon } ((l+1)h) \right| \right| \right| }_h - {\left| \left| \left| \varvec{\varepsilon }(lh) \right| \right| \right| }_h \right) . \end{aligned}$$

Now, by a special version of the discrete Grönwall inequality (Clark 1987), if $z_n$ and $g_n$ are sequences of real numbers (with $g_n \ge 0$), $c \ge 0$ is a nonnegative constant, and if

60 $$\begin{aligned} z_n \le c + \sum _{l=0}^{n-1} g_l z_l, \qquad \text { for all } n \in {\mathbb {N}}, \end{aligned}$$

then

$$\begin{aligned} z_n \le c \prod _{l=0}^{n-1} (1+g_l) \le c \exp \left( \sum _{l=0}^{n-1} g_l \right) , \qquad \text { for all } n \in {\mathbb {N}}. \end{aligned}$$

Application of this inequality to Eq. (59) with $z_n :={\left| \left| \left| \varvec{\varepsilon } ((n+1)h) \right| \right| \right| }_h - {\left| \left| \left| \varvec{\varepsilon }(nh) \right| \right| \right| }_h$, $g_n :=Kh$, and $c :=Kh^{2}$ yields

61 $$\begin{aligned} {\left| \left| \left| \varvec{\varepsilon } ((n+1)h) \right| \right| \right| }_h - {\left| \left| \left| \varvec{\varepsilon }(nh) \right| \right| \right| }_h&\le K(T)h^{2} \exp \left( nKh \right) \end{aligned}$$

62 $$\begin{aligned}&{\mathop {\le }\limits ^{n \le T/h}} K(T)h^2. \end{aligned}$$

By another telescoping sum argument and ${\left| \left| \left| \varvec{\varepsilon }(0) \right| \right| \right| }_h \le Kh^2$, we obtain

63 $$\begin{aligned} {\left| \left| \left| \varvec{\varepsilon }(nh) \right| \right| \right| }_h&{\mathop {=}\limits ^{\text {(tel. sum)}}} \sum _{l=0}^{n-1} \left( {\left| \left| \left| \varvec{\varepsilon }((l+1)h) \right| \right| \right| }_h - {\left| \left| \left| \varvec{\varepsilon }(lh) \right| \right| \right| }_h \right) \nonumber \\&\quad + {\left| \left| \left| \varvec{\varepsilon }(0) \right| \right| \right| }_h \end{aligned}$$

64 $$\begin{aligned}&\quad {\mathop {\le }\limits ^{\text {eq. }(62)}} nK(T)h^2 + Kh^2 \end{aligned}$$

65 $$\begin{aligned}&\quad {\mathop {\le }\limits ^{n \le T/h}} K(T)h + Kh^2 \end{aligned}$$

66 $$\begin{aligned}&\quad \le K(T)h + K h^2, \end{aligned}$$

for all sufficiently small $h>0$. Recalling that $\left\| \varepsilon ^{(0)}(nh) \right\| \le {\left| \left| \left| \varvec{\varepsilon }(nh) \right| \right| \right| }_h$, by Eq. (19), concludes the proof. $\square$

## Calibration of credible intervals

In PN, one way to judge calibration of a Gaussian output ${\mathcal {N}}(m,V)$ is to check whether the implied 0.95 credible interval $[m-2\sqrt{V},m+2\sqrt{V}]$ contracts at the same rate as the convergence rate of the posterior mean to the true quantity of interest. For the filter, this would mean that the rate of contraction of $\max _n \sqrt{P_{00}(nh)}$ should contract at the same rate as $\max _{n \in [T/h + 1]} \Vert \varepsilon ^{(0)}(nh) \Vert$ (recall its rates from Theorem 14). Otherwise, for a higher or lower rate of the interval it would eventually be under- or overconfident, as $h \rightarrow 0$. The following proposition shows—in light of the sharp bound Eq. (58) on the global error—that the credible intervals are well calibrated in this sense if $p \in [1,\infty ]$.

### Theorem 15

Under Assumption 2 and for $R \equiv K h^p$, $p \in [0,\infty ]$, as well as sufficiently small $h>0$,

67 $$\begin{aligned} \max _{n \in [T/h + 1]} P_{00}^- ( n h )&\le K(T)h^{(p+1) \wedge 2}, \qquad \text {and} \end{aligned}$$

68 $$\begin{aligned} \max _{n \in [T/h + 1]} P_{00} ( n h )&\le K(T)h^{(p+1) \wedge 2}. \end{aligned}$$

### Proof

See Appendix J. $$\square $$

## Numerical experiments

In this section, we empirically assess the following hypotheses:

(i) the worst-case convergence rates from Theorem 14 hold not only for $q=1$ but also for $q \in \{2,3\}$ (see Sect. 9.1),
(ii) the convergence rates of the credible intervals from Theorem 15 hold true (see Sect. 9.2), and
(iii) Assumption 4 is necessary to get these convergence rates (see Sect. 9.3).

The three hypotheses are all supported by the experiments. These experiments are subsequently discussed in Sect. 9.4. Appendix D contains an additional experiment illustrating the convergence rates for the state misalignment $\delta$ from Lemma 11.

### Global convergence rates for $q \in \{1,2,3\}$

We consider the following three test IVPs: Firstly, the following linear ODE

69 $$\begin{aligned} {\dot{x}}(t)&= \varLambda x(t),\ \forall t \in [0,10], \nonumber \\ \text { with } \varLambda&= \begin{pmatrix} 0 &{} -\pi \\ \pi &{} 0 \end{pmatrix} \text { and } x(0) = \left( 0 , 1 \right) ^{\intercal }, \end{aligned}$$

and has the harmonic oscillator

70 $$\begin{aligned} x(t) = e^{t\varLambda } x(0) = \begin{pmatrix} -\sin (t \pi )&\cos (t \pi ) \end{pmatrix}^{\intercal } \end{aligned}$$

as a solution. Secondly, the logistic equation

71 $$\begin{aligned} \dot{x}(t)&= \lambda _0 x(t)\left( 1 - x(t)/ \lambda _1 \right) , \ \forall t \in [0,1.5], \nonumber \\&\quad \text { with } (\lambda _0,\lambda _1) = (3,1) \text { and } x(0) = 0.1, \end{aligned}$$

which has the logistic curve

72 $$\begin{aligned} x(t) = \frac{\lambda _1 \exp (\lambda _0 t) x(0)}{\lambda _1 + x(0) (\exp (\lambda _0 t) - 1) }. \end{aligned}$$

**Fig. 1:**
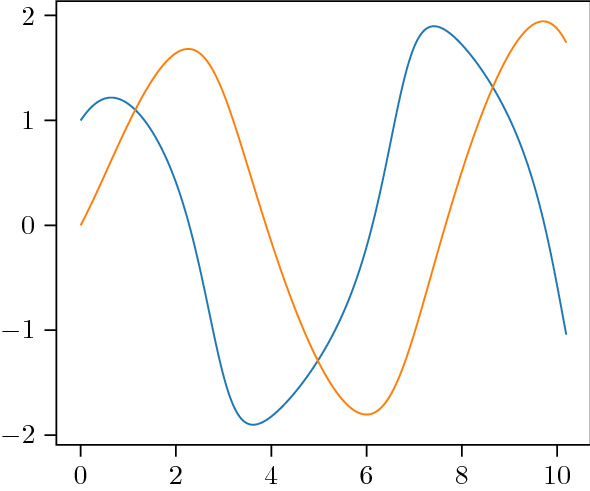
True solution of the FitzHugh–Nagumo model, Eq. (73); $x_1$ in blue and $x_2$ in orange

**Fig. 2:**
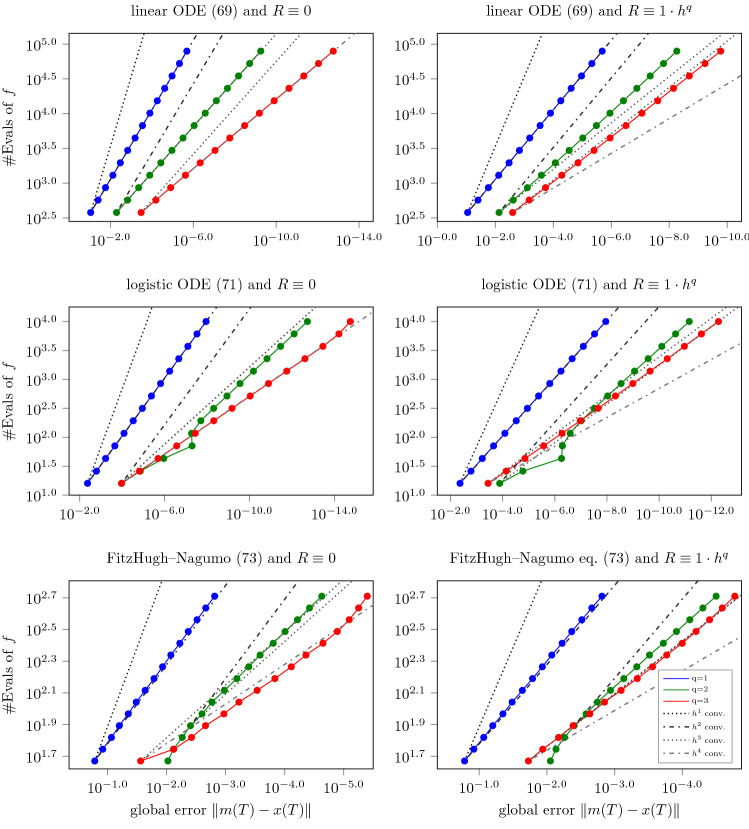
Work-precision diagrams for the Gaussian ODE filter with *q*-times IBM prior, for $q \in \{1,2,3\}$, applied to the linear Eq. (71), logistic ODE Eq. (69) and the FitzHugh–Nagumo model. The number of function evaluations (# Evals of *f*), which is inversely proportional to the step size *h*, is plotted in color against the logarithmic global error at the final time *T*. The (dash-)dotted gray lines visualize idealized convergence rates of orders one to four. The left and right columns employ the minimal $R\equiv 0$ and maximal measurement variance $R \equiv K_R h^q$ ($K_R = 1$) which are permissible under Assumption 4

And, thirdly, the FitzHugh–Nagumo model

73 $$\begin{aligned} \begin{pmatrix} x_1(t) \\ x_2(t) \end{pmatrix} = \begin{pmatrix} x_1(t) - \frac{x_1(t)}{3} - x_2(t) \\ \frac{1}{\tau } \left( x_1(t) + a - bx_2(t) \right) , \end{pmatrix}, \forall t \in [0,10] \end{aligned}$$

with $(a,b,c) = (0.08, 0.07, 1.25)$ and $x(0) = (1,0)$ which does not have a closed-form solution. Its solution, which we approximate by Euler’s method with a step size of $h=10^{-6}$ for the below experiments, is depicted in Fig. 1. We numerically solve these three IVPs with the Gaussian ODE filter for multiple step sizes $h>0$ and with a *q*-times IBM prior (i.e., $\theta = 0$ in Eq. (5)) for $q \in \{1,2,3\}$ and scale $\sigma =20$. As a measurement model, we employ the minimal $R \equiv 0$ and maximal measurement variance $R \equiv K_R h^q$ (for $h \le 1$) which are permissible under Assumption 4 whose constant $K>0$ is denoted explicitly by $K_R$ in this section. The resulting convergence rates of global errors $\left\| m(T) - x(T) \right\|$ are depicted in a work-precision diagram in Fig. 2; cf. Hairer et al. (1987, Chapter II.1.4) for such diagrams for Runge–Kutta methods. Now, recall from Theorem 14 that, for $q=1$, the global truncation error decreases at a rate of at least $h^q$ in the worst case. Figure 2 shows that these convergence rates of *q*th order hold true in the considered examples for values of up to $q=3$ if $R \equiv 0$ and, for values of up to $q=3$. In the case of $R \equiv 0$, even $(q+1)$th order convergence rates appear to hold true for all three ODEs and $q \in \{1,2,3\}$. Note that it is more difficult to validate these convergence rates for $q=4$, for all three test problems and small $h>0$, since numerical instability can contaminate the analytical rates.

### Calibration of credible intervals

**Fig. 3:**
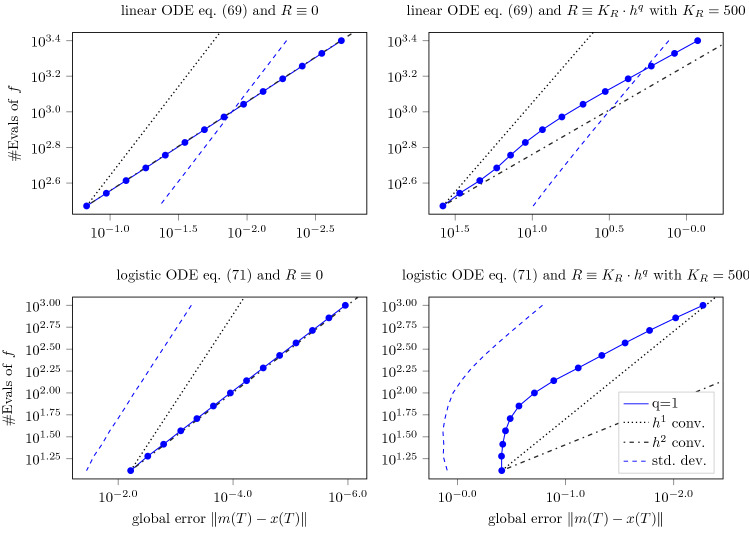
Work-precision diagrams for the Gaussian ODE filter with *q*-times IBM prior, for $q=1$, applied to the linear Eq. (69) and logistic ODE Eq. (71) in the upper and lower row, respectively. The number of function evaluations (# Evals of *f*), which is inversely proportional to the step size *h*, is plotted in color against the logarithmic global error at the final time *T*. The (dash-)dotted gray lines visualize idealized convergence rates of orders one and two. The dashed blue lines show the posterior standard deviations calculated by the filter. The left and right columns, respectively, employ the minimal $R\equiv 0$ and maximal measurement variance $R \equiv K_R h^q$ ($K_R = 5.00 \times 10^3$) which are permissible under Assumption 4

To demonstrate the convergence rates of the posterior credible intervals proved in Theorem 15, we now restrict our attention to the case of $q=1$, that was considered therein. As in Sect. 9.1, we numerically solve the IVPs eqs. (69) and (71) with the Gaussian ODE filter with a once IBM prior with fixed scale $\sigma =1$. We again employ the minimal $R \equiv 0$ and maximal measurement variance $R \equiv K_R h^q$ (for $h \le 1$) which are permissible under Assumption 4 as a measurement model. Figure 3 depicts the resulting convergence rates in work-precision diagrams. As the parallel standard deviation (std. dev.) and $h^1$ convergence curves show, the credible intervals asymptotically contract at the rate of $h^1$ guaranteed by Theorem 15. In all four diagrams of Fig. 3, the global error shrinks at a faster rate than the width of the credible intervals. This is unsurprising for $R \equiv 0$ as we have already observed convergence rates of $h^{q+1}$ in this case. While this effect is less pronounced for $R \equiv K_R h^q$, it still results in underconfidence as $h \rightarrow 0$. Remarkably, the shrinking of the standard deviations seems to be ‘adaptive’ to the numerical error—by which we mean that, as long as the numerical error hardly decreases (up to $10^{1.75}$ evaluations of *f*), the standard deviation also stays almost constant, before adopting its $h^1$ convergence asymptotic (from $\approx 10^{2.00}$).

### Necessity of Assumption 4

**Fig. 4:**
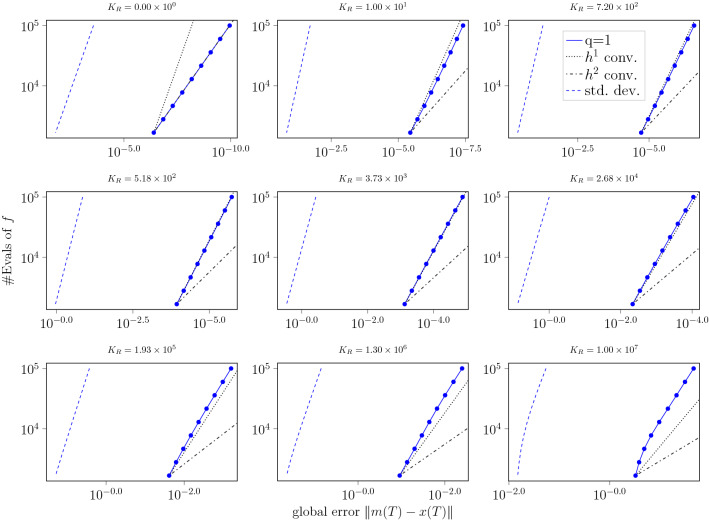
Work-precision diagrams for the Gaussian ODE filter with *q*-times IBM prior, for $q=1$ and $R\equiv K_R h^{1/2}$, applied to the logistic ODE Eq. (71) for increasing values of $K_R$. The number of function evaluations (# Evals of *f*), which is inversely proportional to the step size *h*, is plotted in blue against the logarithmic global error at the final time *T*. The (dash-)dotted gray lines visualize idealized convergence rates of orders one and two. The dashed blue lines show the posterior standard deviations calculated by the filter

Having explored the asymptotic properties under Assumption 4 in Sects. 9.1 and 9.2, we now turn our attention to the question of whether this assumption is necessary to guarantee the convergence rates from Theorems 14 and 15. This question is of significance, because Assumption 4 is weaker than the $R \equiv 0$ assumption of the previous theoretical results (i.e., Proposition 1 and Theorem 1 in Schober et al. (2019)) and it is not self-evident that it cannot be further relaxed. To this end, we numerically solve the logistic ODE Eq. (71) with the Gaussian ODE filter with a once IBM prior with fixed scale $\sigma = 1$ and measurement variance $R \equiv K_R h^{1/2}$, which is impermissible under Assumption 4, for increasing choices of $K_R$ from $0.00 \times 10^0$ to $1.00 \times 10^7$. In the same way as in Fig. 3, the resulting work-precision diagrams are plotted in Fig. 4.

In contrast to the lower left diagram in Fig. 3, which presents the same experiment for $R \equiv K_R h^q$ (the maximal measurement variance permissible under Assumption 4), the rate of $h^2$, that is again observed for $K_R = 0$ in the first diagram, is already missed for $K_R = 1.00 \times 10^0$ in the second diagram. With growing constants, the convergence rates of the actual errors as well as the expected errors (standard deviation) decrease from diagram to diagram. In the center diagram with $K_R=3.73 \times 10^3$, the rates are already slightly worse than the $h^1$ convergence rates guaranteed by Theorems 14 and 15 under Assumption 4, whereas, for $K_R = 5.00 \times 10^3$, the convergence rates in the lower left plot of Fig. 3 were still significantly better than $h^1$. For the greater constants up to $K_R = 1.00 \times 10^7$, the rates even become significantly lower. Notably, as in the lower right diagram of Fig. 3, the slope of the standard deviation curve matches the slope of the global error curve, as can be seen best in the lower right subfigure—thereby asymptotically exhibiting neither over- nor underconfidence. These experiments suggest that the convergence rates from Theorems 14 and 15 do not hold in general for $R \equiv K_R h^{1/2}$. Hence, it seems likely that Assumption 4 is indeed necessary for our results and cannot be further relaxed without lowering the implied worst-case convergence rates.

### Discussion of experiments

Before proceeding to our overall conclusions, we close this section with a comprehensive discussion of the above experiments. First and foremost, the experiments in Sect. 9.1 suggest that Theorem 14, the main result of this paper, might be generalizable to $q \in \{2,3\}$ and potentially even higher $q \in {\mathbb {N}}$—although unresolved issues with numerical instability for small step sizes prevent us from confidently asserting that these theoretical results would hold in practice for $q \ge 4$. Moreover, we demonstrated the contraction rates of the posterior credible intervals from Theorem 15 and evidence for the necessity of Assumption 4 in Sects. 9.3 and 9.2. The asymptotics revealed by these experiments can be divided by the employed measurement model into three cases: the zero-noise case $R \equiv 0$, the permissible nonzero case $R \le K_R h^q$ (under Assumption 4) and the non-permissible case $R \nleq K_R h^q$. First, if $R \equiv 0$, the diagrams in the left column of Fig. 2 reaffirm the $h^{q+1}$ convergence reported for $q \in \{1,2\}$ in Schober et al. (2019, Figure 4) and extend them to $q=3$ (see Sect. 10 for a discussion on why we expect the above global convergence proofs to be extensible to $q \ge 2$)

The contraction rates of the credible intervals, for $q = 1$, appear to be asymptotically underconfident in this case as they contract faster than the error. This underconfidence is not surprising in so far as the posterior standard deviation is a worst-case bound for systems modeled by the prior, while the convergence proofs require smoothness of the solution of one order higher than sample paths from the prior. This is a typical result that highlights an aspect known to, but on the margins of classic analysis: The class of problems for which the algorithm converges is rougher than the class on which convergence order proofs operate. How to remedy such overly cautious UQ remains an open research question in PN as well as classical numerical analysis.

Secondly, in the case of $R>0$, as permissible under Assumption 4, the convergence rates are slightly reduced compared to the case $R \equiv 0$, exhibiting convergence between $h^q$ and $h^{q+1}$. The asymptotic underconfidence of the credible intervals, however, is either reduced or completely removed as depicted in the right column of Fig. 3. Thirdly, in the final case of an impermissibly large $R>0$, the $h^q$ convergence speed guaranteed by Theorem 14 indeed does not necessarily hold anymore—as depicted in Fig. 4. Note, however, that even then the convergence rate is only slightly worse than $h^q$. The asymptotic UQ matches the observed global error in this case, as the parallel standard deviation and the $h^1$ curves in all but the upper left $R \equiv 0$ diagram show.

Overall, the experiments suggest that, in absence of statistical noise on *f*, a zero-variance measurement model yields the best convergence rates of the posterior mean. Maybe this was expected as, in this case, *R* only models the inaccuracy from the truncation error, that ideally should be treated adaptively (Kersting and Hennig 2016, Section 2.2). The convergence rates of adaptive noise models should be assessed in future work. As the observed convergence rates in practice sometimes outperform the proved worst-case convergence rates, we believe that an average-case analysis of the filter in the spirit of Ritter (2000) may shed more light upon the expected practical performance. Furthermore, it appears that the UQ becomes asymptotically accurate as well as adaptive to the true numerical error as soon as the $R>0$ is large enough. This reinforces our hope that these algorithms will prove useful for IVPs when *f* is estimated itself (Hennig et al. 2015, Section 3(d)), thereby introducing a $R>0$.

## Conclusions

We presented a worst-case convergence rate analysis of the Gaussian ODE filter, comprising both local and global convergence rates. While local convergence rates of $h^{q+1}$ were shown to hold for all $q \in {\mathbb {N}}$, IBM and IOUP prior as well as any noise model $R \ge 0$, our global convergence results is restricted to the case of $q=1$, IBM prior and fixed noise model $R \equiv Kh^p$ with $p \in [1,\infty ]$. While a restriction of the noise model seems inevitable, we believe that the other two restrictions can be lifted: In light of Theorem 8, global convergence rates for the IOUP prior might only require an additional assumption that ensures that all possible data sequences $\{y(nh);n=1,\dots ,T/h\}$ (and thereby all possible *q*th-state sequences $\{m^{(q)}(nh);n=0,\dots ,T/h\}$) remain uniformly bounded (see discussion in Remark 2). For the case of $q \ge 2$, it seems plausible that a proof analogous to the presented one would already yield global convergence rates of order $h^q$,^3^ as suggested for $q \in \{2,3\}$ by the experiments in Sect. 9.1.

The orders of the predictive credible intervals can also help to intuitively explain the threshold of $p=1$ (or maybe more generally: $p=q$; see Fig. 2) below which the performance of the filter is not as good, due to Eqs. (45)–(49): According to Kersting and Hennig (2016, Equation (20)), the ‘true’ (push-forward) variance on *y*(*t*) given the predictive distribution ${\mathcal {N}}(m^-(t),P^-(t))$ is equal to the integral of $f f^{\intercal }$ with respect to ${\mathcal {N}}(m^-(t),P^-(t))$, whose maximum over all time steps, by Eq. (67), has order ${\mathcal {O}}(h^{\frac{p+1}{2} \wedge 1})$ if $ff^{\intercal }$ is globally Lipschitz—since $P^-(t)$ enters the argument of the integrand $f f^{\intercal }$, after a change of variable, only under a square root. Hence, the added ‘statistical’ noise *R* on the evaluation of *f* is of lower order than the accumulated ‘numerical’ variance $P^-(t)$ (thereby preventing numerical convergence) if and only if $p<1$. Maybe this, in the spirit of Hennig et al. (2015, Subsection 3(d)), can serve as a criterion for vector fields *f* that are too roughly approximated for a numerical solver to output a trustworthy result, even as $h \rightarrow 0$.

Furthermore, the competitive practical performance of the filter, as numerically demonstrated in Schober et al. (2019, Section 5), might only be completely captured by an average-case analysis in the sense of Ritter (2000), where the average error is computed with respect to some distribution *p*(*f*), i.e., over a distribution of ODEs. To comprehend this idea, recall that the posterior filtering mean is the Bayes estimator with minimum mean squared error in linear dynamical systems with Gauss–Markov prior (as defined by the SDE Eq. (2)), i.e., when the data is not evaluations of *f* but real i.i.d. measurements, as well as in the special case of $\dot{x}(t) = f(t)$, when the IVP simplifies to a quadrature problem—see Solak et al. (2003) and O’Hagan (1991, Section 2.2), respectively. In fact, the entire purpose of the update step is to correct the prediction in the (on average) correct direction, while a worst-case analysis must assume that it corrects in the worst possible direction in every step—which we execute by the application of the triangle inequality in Eq. (28) resulting in a worst-case upper bound that is the sum of the worst-case errors from prediction and update step. An analysis of the probabilities of ‘good’ vs. ‘bad’ updates might therefore pave the way for such an average-case analysis in the setting of this paper. Since, in practice, truncation errors of ODE solvers tend to be significantly smaller than the worst case—as mirrored by the experiments in Section 9—such an analysis might be useful for applications.

Lastly, we hope that the presented convergence analysis can lay the foundations for similar results for the novel ODE filters (extended KF, unscented KF, particle filter) introduced in Tronarp et al. (2019), and can advance the research on uncertainty-aware likelihoods for inverse problems by ODE filtering (Kersting et al. 2020, Section 3).

## Figures and Tables

**Fig. 5:**
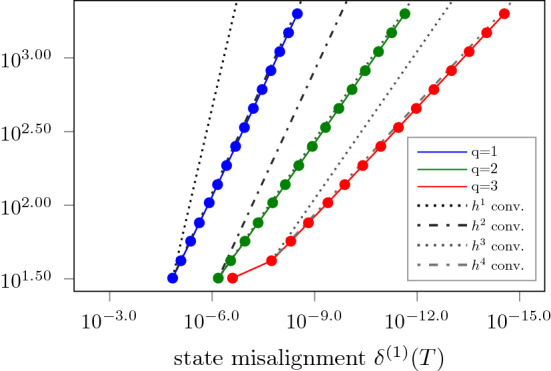
Work-precision diagram plotting the number of function evaluations (# Evals of *f*) against the final state misalignment $\delta ^{(1)}(T)$ on the Riccati Eq. (85); cf. Fig. 2

## Derivation of *A* and *Q*

As derived in Särkkä (2006, Section 2.2.6) the solution of the SDE Eq. (2), i.e.,

74 $$\begin{aligned} \mathrm {d}{{\varvec{X}}(t)}&= \begin{pmatrix} \mathrm {d}{X^{(0)}(t)} \\ \vdots \\ \mathrm {d}{X^{(q-1)}(t)} \\ \mathrm {d}{X^{(q)}(t)} \end{pmatrix} \nonumber \\&= \underbrace{\begin{pmatrix} 0 &{} 1 &{} 0 \dots &{} 0 \\ \vdots &{} \ddots &{} \ddots &{} 0 \\ \vdots &{} &{} \ddots &{} 1 \\ c_0 &{} \dots &{} \dots &{} c_q \end{pmatrix}}_{=:F} \underbrace{\begin{pmatrix} {X^{(0)}(t)} \\ \vdots \\ {X^{(q-1)}(t)} \\ {X^{(q)}(t)} \end{pmatrix}}_{= {{\varvec{X}} (t) }} \ \mathrm {d}t + \underbrace{\begin{pmatrix} 0 \\ \vdots \\ 0 \\ \sigma \end{pmatrix}}_{=:L}\ \mathrm {d}B{(t)}, \end{aligned}$$

where we omitted the index *j* for simplicity, is a Gauss–Markov process with mean *m*(*t*) and covariance matrix *P*(*t*) given by

75 $$\begin{aligned} m(t) = A(t) m(0), \qquad P(t) = A(t)P(0) A(t)^{\intercal } + Q{(t)}, \end{aligned}$$

where the matrices $A,\ Q \in {\mathbb {R}}^{(q+1)\times (q+1)}$ are explicitly defined by

76 $$\begin{aligned} A(t) =&\exp (tF), \end{aligned}$$

77 $$\begin{aligned} Q{(t)} :=&\int _0^t \exp (F({t}-\tau )){L L^{\intercal }} \exp (F({t}-\tau ))^{\intercal }\ \mathrm {d}\tau . \end{aligned}$$

Parts of the following calculation can be found in Magnani et al. (2017). If we choose $c_0, \dots , c_{q-1} = 0$ and $c_q= - \theta$ (for $\theta \ge 0$) in Eq. (74) the unique strong solution of the SDE is a *q*-times IOUP, if $\theta >0$, and a *q*-times IBM, if $\theta =0$; see, e.g., Karatzas and Shreve (1991, Chapter 5: Example 6.8). By Eq. (77) and

78 $$\begin{aligned} { \begin{pmatrix} (tF)^k \end{pmatrix}_{i,j} = t^k \left[ {\mathbb {I}}_{j-i=k} + (- \theta )^{k+i-q} {\mathbb {I}}_{\{j=q,\ i+k\ge q\}} \right] , } \end{aligned}$$

it follows that

79 $$\begin{aligned} A(t)_{ij}&= \begin{pmatrix} \sum \nolimits _{k=0}^{\infty } \frac{(tF)^k}{k!} \end{pmatrix}_{i,j} \nonumber \\&= {\left\{ \begin{array}{ll} {\mathbb {I}}_{i\le j} \frac{t^{j-i}}{(j-i)!}, &{} \text{ if } j\ne q, \\ \frac{1}{(-\theta )^{q-i}} \sum \nolimits _{k=q-i}^{\infty } \frac{(- \theta t)^k}{k!} , &{} \text{ if } j=q, \end{array}\right. } \nonumber \\&{= {\left\{ \begin{array}{ll} {\mathbb {I}}_{i\le j} \frac{t^{j-i}}{(j-i)!}, &{} \text{ if } j\ne q, \\ \frac{t^{q-i}}{(q-i)!} - \theta \sum \nolimits _{k=q+1-i}^{\infty } \frac{(-\theta )^{k+i-q-1} t^k}{ k! } , &{} \text{ if } j=q. \end{array}\right. } } \end{aligned}$$

Analogously, it follows that

80 $$\begin{aligned}&\exp (F(t-\tau )) \nonumber \\&\quad = {\left\{ \begin{array}{ll} {\mathbb {I}}_{i\le j} \frac{(t-\tau )^{j-i}}{(j-i)!}, &{} \text{ if } j\ne q, \\ \frac{(t-\tau )^{q-i}}{(q-i)!} - \theta \sum \nolimits _{k=q+1-i}^{\infty } \frac{(-\theta )^{k+i-q-1} (t-\tau )^k}{ k! } , &{} \text{ if } j=q, \end{array}\right. }. \end{aligned}$$

If we insert Eq. (80) into Eq. (77), then we obtain, by the sparsity of *L*, that

81 $$\begin{aligned}&{Q(t)}_{ij} \nonumber \\&\quad = \frac{\sigma ^2}{{(-\theta )}^{2q-i-j}} \int _0^t \left( \sum _{k=q-i}^{\infty } \frac{(-\theta \tau )^k}{k!} \right) \left( \sum _{l=q-j}^{\infty } \frac{(-\theta \tau )^l}{l!} \right) \ \mathrm {d}\tau , \end{aligned}$$

and the dominated convergence theorem (with dominating function $\tau \mapsto e^{2\theta \tau }$) yields

82 $$\begin{aligned} Q(t)_{ij}&= \frac{\sigma ^2}{(-\theta )^{2q-i-j}} \sum _{k=q-i}^{\infty } \sum _{l=q-j}^{\infty } \int _0^t \frac{(-\theta \tau )^{k+l}}{k!l!} \ \mathrm {d}\tau \nonumber \\&= \frac{\sigma ^2}{(-\theta )^{2q-i-j}} \sum _{k=q-i}^{\infty } \sum _{l=q-j}^{\infty } (-\theta )^{k+l} \frac{ t^{k+l+1}}{(k+1+l)k!l!}. \end{aligned}$$

Now, by extracting the first term and noticing that the rest of the series is in $\varTheta (t^{2q+2-i-j})$, it follows that

83 $$\begin{aligned} Q(t)_{ij}&= \sigma ^2 \frac{t^{2q+1-i-j}}{(2q+1-i-j)(q-i)!(q-j)!} \nonumber \\&\quad + \varTheta \left( t^{2q+2-i-j}\right) . \end{aligned}$$

## Extension to *x* with dependent dimensions

The algorithm in Sect. 2.2 employs a prior ${\varvec{X}}$ with independent dimensions ${\varvec{X}}_j = \left( X_j^{(0)}, \dots , X_j^{(q)} \right) ^ \intercal$, $j \in [d]$, by Eq. (2). While this constitutes a loss of generality for our new theoretical results, which do not immediately carry over to the case of *x* with dependent dimensions, it is not a restriction to the class of models the algorithm can employ. To construct such a prior ${\varvec{X}}$, we first stack its dimensions into the random vector ${\varvec{X}} = (\varvec{X}_0^{\intercal },\dots ,{\varvec{X}}_{d-1}^{\intercal })^{\intercal }$, choose symmetric positive semi-definite matrices $K_x,K_{\varepsilon } \in {\mathbb {R}}^{d \times d}$, and define, using the Kronecker product $\otimes$, its law according to the SDE

84 $$\begin{aligned} \mathrm {d}{\varvec{X}}(t) = \left[ K_x \otimes F \right] {\varvec{X}}(t)\, \mathrm {d}t + \left[ K_{\varepsilon } \otimes L \right] \, \mathrm {d}B(t), \end{aligned}$$

with initial condition $${\varvec{X}}(0) \sim {\mathcal {N}}(m(0),P(0))$$, mean $$m(0) \in {\mathbb {R}}^{d(q+1)}$$ and covariance matrix $$P(0) \in {\mathbb {R}}^{d(q+1) \times d(q+1)}$$, as well as an underlying *d*-dimensional Brownian motion *B* (independent of $${\varvec{X}}(0)$$). Now, insertion of $$K_x \otimes F$$ and $${K_{\varepsilon }} \otimes L$$ for *F* and *L* into Eq. (77) yields new predictive matrices $${{\tilde{A}}}$$ and $${{\tilde{Q}}}$$. If we now choose $$K_x = I_d $$ and $$K_{\varepsilon } = I_d$$, substitute $${{\tilde{A}}}$$ and $${{\tilde{Q}}}$$ for *A* and *Q* in Eqs. (9) and (10), and use the $$d(q+1)$$-dimensional GP $${\varvec{X}}$$ from Eq. (84) with $$m(0) \in \mathbb R^{d(q+1)}$$ and $$P(0) \in {\mathbb {R}}^{d(q+1) \times d(q+1)}$$ as a prior, we have equivalently defined the version of Gaussian ODE filtering with independent dimensions from Sect. 2.2. If we, however, choose different symmetric positive semi-definite matrices for $$K_x$$ and $$K_{\varepsilon }$$, we introduce, via $${{\tilde{A}}}$$ and $${{\tilde{Q}}}$$, a correlation in the development of the solution dimensions $$(x_0,\dots ,x_{d-1})^{\intercal }$$ as well as the error dimensions $$(\varepsilon _0,\dots ,\varepsilon _d)^{\intercal }$$, respectively. Note that, while $$K_{\varepsilon }$$ plays a similar role as $$C^h$$ in Conrad et al. (2017, Assumption 1) in correlating the numerical errors, the matrix $$K_x$$ additionally introduces a correlation of the numerical estimates, that is *m*, along the time axis. Even more flexible correlation models (over all modeled derivatives) can be employed by inserting arbitrary matrices (of the same dimensionality) for $$K_x \otimes F$$ and $${K_{\varepsilon }} \otimes L$$ in Eq. (84), but such models seem hard to interpret. For future research, it would be interesting to examine whether such GP models with dependent dimensions are useful in practice. There are first publications (Xiaoyue et al. 2018; Gessner et al. 2019) on this topic for integrals, but not yet for ODEs.

## Illustrative example

To illustrate the algorithm defined in Sect. 2.2, we apply it to a special case of the Riccati equation (Davis 1962, p. 73)

85 $$\begin{aligned} \frac{\mathrm {d}x}{\mathrm {d}t} (t) = f(x(t))&= - \frac{(x(t))^3}{2}, \quad x(0) = 1, \end{aligned}$$

86 $$\begin{aligned} \Big (\text {solution: } x(t)&= (t+1)^{-1/2} \Big ), \end{aligned}$$

with step size $h=0.1$, measurement noise $R=0.0$ (for simplicity) as well as prior hyperparameters $q=1$, $\sigma ^2 = 10.0$ and $c_i = 0$ for all $i \in [q+1]$ (recall Eq. (2)), i.e., with a 1-times integrated Brownian motion prior whose drift and diffusion matrices are, by Eq. (8), given by

87 $$\begin{aligned} A(h) = \begin{pmatrix} 1 &{} h \\ 0 &{} 1 \end{pmatrix}, \qquad Q(h) = \begin{pmatrix} 1/300 &{} 1/20 \\ 1/20 &{} 1 \end{pmatrix}. \end{aligned}$$

As the ODE Eq. (85) is one-dimensional (i.e., $d=1$), the dimension index $j \in [d]$ is omitted in this section. Since the initial value and derivative are certain at $x(0) = 1$ and $\dot{x}(0) = f(x_0) = -1/2$, our prior GP is initialized with a Dirac distribution (i.e., ${\varvec{X}}(0) = ( X^{(0)}(0), X^{(1)}(0) )^{\intercal } \sim \delta _{(x_0,f(x_0))} = \delta _{(1,-1/2)}$). Therefore, ${\varvec{m}}(0) = (1,-1/2)^{\intercal }$ and $P(0) = 0 \in {\mathbb {R}}^{2 \times 2}$ for the initial filtering mean and covariance matrix. Now, the Gaussian ODE Filter computes the first integration step by executing the prediction step Eqs. (9) and (10)

88 $$\begin{aligned} {\varvec{m}}^-(h)&= A(h) {\varvec{m}}^-(0) \nonumber \\&= \left( m^{(0)}(0) + h m^{(1)}(0),m^{(1)}(0) \right) ^{\intercal } \nonumber \\&= \left( 19/20, -1/2 \right) ^{\intercal }, \qquad \text {and} \end{aligned}$$

89 $$\begin{aligned} P^-(h)&= 0+Q(h) = \begin{pmatrix} 1/300 &{} 1/20 \\ 1/20 &{} 1 \end{pmatrix}. \end{aligned}$$

Note that, for all $i \in [q+1]$, $m^{-,(i)}(h)$ is obtained by a $(q-i)$th-order Taylor expansion of the state ${\varvec{m}}(0) = (x_0,f(x_0))^{\intercal } \in {\mathbb {R}}^{q+1}$. Based on this prediction, the data is then generated by

90 $$\begin{aligned} y(h)&= f\left( m^{-,(0)}(h)\right) {\mathop {=}\limits ^{\text {eq. }(88)}} f(19/20) \nonumber \\&{\mathop {=}\limits ^{\text {eq. }(85)}} -6859/16000 \end{aligned}$$

with variance $R = 0.0$. In the subsequent update step Eqs. (9) and (11) to (13), a Bayesian conditioning of the predictive distribution Eqs. (88) and (89) on this data is executed:

91 $$\begin{aligned} \varvec{\beta } (h)&= \left( \beta ^{(0)}(h), \beta ^{(1)}(h) \right) ^{\intercal } \nonumber \\&= \left( \frac{P^-(h)_{01}}{(P^-(h))_{11} + R}, \frac{P^-(h)_{11}}{(P^-(h))_{11} + R} \right) ^{\intercal } \nonumber \\&{\mathop {=}\limits ^{\text {eq. }(89)}} \left( \frac{1}{20}, 1 \right) ^{\intercal }, \end{aligned}$$

92 $$\begin{aligned} r(h)&= y(h) - m^{-,(1)}(h) \nonumber \\&{\mathop {=}\limits ^{\text {eqs. }(88), (90)}} -6859/16000 + 1/2 \nonumber \\&= 1141/16000 , \end{aligned}$$

93 $$\begin{aligned} {\varvec{m}}(h)&{\mathop {=}\limits ^{\text {eq. }(9)}} \begin{pmatrix} m^{-,(0)}(h) + \beta ^{(0)}(h) r(h) \\ m^{-,(1)}(h) + \beta ^{(1)}(h) r(h) \end{pmatrix} \nonumber \\&{\mathop {=}\limits ^{\text {eqs. }(88), (91),(92)}} \begin{pmatrix} 305141/320000 \\ -6859/16000 \end{pmatrix}, \end{aligned}$$

which concludes the step from 0 to *h*. The next step $h \rightarrow 2h$ starts with computing $m^{-,(i)}(2h)$ by a $(q-i)$th-order Taylor expansion of the *i*th state $m^{(i)}(h)$, for all $i \in [q+1]$. Note that, now, there is a nonzero *state misalignment* (recall Eq. (25)):

94 $$\begin{aligned} \delta ^{(1)}(h)&\overset{eq. (25)}{=} \left| m^{(1)}(h) - f\left( m^{(0)}(h) \right) \right| \end{aligned}$$

95 $$\begin{aligned}&= \left| - \frac{6859}{16000} - \frac{1}{2} \left( \frac{305141}{320000} \right) ^3 \right| \end{aligned}$$

96 $$\begin{aligned}&\approx 0.00485 >0 \end{aligned}$$

which confirms the exposition on the possibility of $\delta ^{(i)} > 0$ from Sect. 4. Note that $\delta$ tends to increase with *R*; e.g., if $R=1.0$ in the above example, then $\delta ^{(1)}(h) \approx 0.03324$.

## Experiment: global convergence of state misalignments $\delta$

Figure 5 depicts the global convergence of the state misalignment $\delta ^{(1)}(T)$ in the above example Eq. (85), as detailed in Appendix C, for $q \in \{1,2,3\}$. The plotting is analogous to Fig. 2. The resulting convergence rates of $h^{q+1}$ confirm Lemma 11 and suggest that it may also be generalizable to $q \in \{2,3,\dots \}$.

## Proof of Eq. (23)

We prove the stronger statement

97 $$\begin{aligned} \varPhi _t^{(i+1)}(a) = f^{(i)} \left( \varPhi _t^{(0)}(a) \right) , \end{aligned}$$

from which Eq. (23) follows by inserting $t=0$ and $\varPhi _0^{(0)}(a) = a$. Hence, it remains to show Eq. (97).

### Proof

*(of Eq. *(97)) By induction over $i \in \{0,\dots ,q\}$. The base case $(i=0)$ is obtained using the fundamental theorem of calculus and $f^{(1)} = f$: $\varPhi _t^{(1)}(a) = f\left( \varPhi _t^{(0)}(a) \right) = f^{(1)}\left( \varPhi _t^{(0)}(a) \right) .$ For the inductive step $(i-1) \rightarrow i$, we conclude (using the inductive hypothesis (IH), the chain rule (CR), the base case (BC) and $f^{(i)} = \nabla _x f^{(i-1)} \cdot f$) that

98 $$\begin{aligned} \varPhi _t^{(i+1)}(a)&= \frac{\mathrm {d}}{\mathrm {d}t} \varPhi _t^{(i)}(a) \nonumber \\&{\mathop {=}\limits ^{\text {(IH)}}} \frac{\mathrm {d}}{\mathrm {d}t} f^{(i-1)}\left( \varPhi _t^{(0)}(a) \right) \nonumber \\&{\mathop {=}\limits ^{\text {(CR)}}} \nabla _x f^{(i-1)}\left( \varPhi _t^{(0)}(a) \right) \frac{\mathrm {d}}{\mathrm {d}t} \varPhi _t^{(0)}(a) \nonumber \\&= \nabla _x f^{(i-1)}\left( \varPhi _t^{(0)}(a) \right) \cdot f \left( \varPhi _t^{(0)}(a) \right) \nonumber \\&= \left[ \nabla _x f^{(i-1)} \cdot f \right] \left( \varPhi _t^{(0)}(a) \right) \nonumber \\&{\mathop {=}\limits ^{\text {(BC)}}} f^{(i)} \left( \varPhi _t^{(0)}(a) \right) . \end{aligned}$$

$$\square $$

## Proof of Lemma 5

### Proof

Again, w.l.o.g. $d=1$. Recall that, by Eq. (13), *r* is implied by the values of $m^{-,(0)}$ and $m^{-,(1)}$. By insertion of

99 $$\begin{aligned}&m^{-,(i)}((n+1)h) \nonumber \\&\quad = \sum _{k=i}^q \frac{h^{k-i}}{(k-i)!} m^{(k)}(nh) + K \theta \left| m^{(q)}(nh) \right| h^{q+1-i} \end{aligned}$$

(due to Eqs. (8) and (14)) into the definition Eq. (13) of $r((n+1)h)$, we obtain the following equality which we then bound by repeated application of the triangle inequality:

100 $$\begin{aligned}&\left| r((n+1)h) \right| \nonumber \\&\quad = \Bigg \vert f\left( \sum _{k=0}^q \frac{h^k}{k!} m^{(k)}(nh) + {K \theta \left| m^{(q)}(nh) \right| h^{q+1} } \right) \nonumber \\&\qquad - \left( \sum _{k=1}^q \frac{h^{k-1}}{(k-1)!} m^{(k)}(nh) + {K \theta \left| m^{(q)}(nh) \right| h^{q}} \right) \Bigg \vert \nonumber \\&\quad \le \Bigg \vert f\left( \sum _{k=0}^q \frac{h^k}{k!} m^{(k)}(nh) + {K \theta \left| m^{(q)}(nh) \right| h^{q+1}} \right) \nonumber \\&\qquad - \left( \sum _{k=1}^q \frac{h^{k-1}}{(k-1)!} m^{(k)}(nh) \right) \Bigg \vert \, + \, {K \theta \left| m^{(q)}(nh) \right| h^{q}} \nonumber \\&\quad {\mathop {\le }\limits ^{\text {eq. }(25)}} I_1(h) + I_2(h) + I_3(h) \nonumber \\&\qquad \qquad + \sum _{k=1}^q \frac{h^{k-1}}{(k-1)!} \delta ^{(k)}(nh) {+} {K \theta \left| m^{(q)}(nh) \right| h^{q}}, \end{aligned}$$

where $I_1$, $I_2$, and $I_3$ are defined and bounded as follows, using Assumption 1 and Lemma 1:

101 $$\begin{aligned} I_1(h)&:=\Bigg \vert f \left( \sum _{k=0}^q \frac{h^k}{k!} m^{(k)}(nh) + {K \theta \left| m^{(q)}(nh) \right| h^{q+1}} \right) \nonumber \\&\quad - f\left( \sum _{k=0}^q \frac{h^k}{k!} \varPhi _0^{(k)}\left( m^{(0)}(nh) \right) \right) \Bigg \vert \nonumber \\&\le L \sum _{k=0}^q \frac{h^k}{k!} \delta ^{(k)}(nh) + {L K \theta \left| m^{(q)}(nh) \right| h^{q+1}}, \end{aligned}$$

102 $$\begin{aligned} I_2(h)&:=\Big \vert f \left( \sum _{k=0}^q \frac{h^k}{k!} \varPhi _0^{(k)}\left( m^{(0)}(nh) \right) \right) - f\left( \varPhi _h^{(0)} \left( m^{(0)}(nh) \right) \right) \Big \vert \nonumber \\&\le L \left| \sum _{k=0}^q \frac{h^k}{k!} \varPhi _0^{(k)}\left( m^{(0)}(nh) \right) - \varPhi _h^{(0)} \left( m^{(0)} (nh) \right) \right| \nonumber \\&{\mathop {\le }\limits ^{\text {eq. }(16)}} Kh^{q+1}, \end{aligned}$$

and

103 $$\begin{aligned} I_3(h)&:=\left| \varPhi _h^{(1)} \left( m^{(0)}(nh) \right) - \sum _{k=1}^q \frac{h^{k-1}}{(k-1)!} \varPhi _0^{(k)} \left( m^{(0)}(nh) \right) \right| \nonumber \\&{\mathop {\le }\limits ^{\text {eq. }(16)}} Kh^q. \end{aligned}$$

Inserting Eqs. (101), (102), and (103) into Eq. (100) (and recalling $\delta ^{(0)}=0$) yields Eq. (31). $\square$

## Proof of Lemma 9

### Proof

Let ${{\tilde{u}}}_0 = u^{*}$ and ${{\tilde{u}}}_n = T_n({{\tilde{u}}}_{n-1})$, for $n \in {\mathbb {N}}$. Then,

104 $$\begin{aligned} d(u^{*},x_n) \le \underbrace{d(u^{*},u_n)}_{\rightarrow 0} + \underbrace{d(u_n,{{\tilde{u}}}_n)}_{=:a_n} + \underbrace{d(\tilde{u}_n,x_n)}_{\rightarrow 0}, \end{aligned}$$

where the last summand goes to zero by

$$\begin{aligned} d({{\tilde{u}}}_n,x_n)&= d\left( (T_n \circ \dots \circ T_1) (u^{*}), (T_n \circ \dots \circ T_1)(x_0)\right) \\&\le {{\bar{L}}}^n d(u^{*},x_0)\ \rightarrow \ 0, \qquad \text { as } n \rightarrow \infty . \end{aligned}$$

Hence, it remains to show that $\lim _{n\rightarrow \infty } a_n = 0$. The ${{\bar{L}}}$-Lipschitz continuity of $T_n$ and the triangle inequality yield that

105 $$\begin{aligned} a_n&= d(T_n(u_{n}), T_n({{\tilde{u}}}_{n-1})) \nonumber \\&\le {{\bar{L}}}\left[ d(u_n, u_{n-1}) + d( u_{n-1}, {{\tilde{u}}}_{n-1}) \right] \nonumber \\&= {{\bar{L}}} a_{n-1} + b_{n-1} , \end{aligned}$$

where $$b_{n} :={{\bar{L}}} d(u_{n+1},u_{n}) \rightarrow 0$$. Now, for all $$m \in {\mathbb {N}}$$, let $$a^{(m)}_0 :=a_0 $$ and $$a^{(m)}_n :={{\bar{L}}}a^{(m)}_{n-1} + b_m$$. By BFT, $$\lim _{n \rightarrow \infty } a^{(m)}_n = b_m / (1-{{\bar{L}}})$$. Since, for all $$m \in {\mathbb {N}}$$, $$a_n \le a^{(m)}_n$$ for sufficiently large *n*, it follows that

106 $$\begin{aligned} 0 \le \limsup _{n \rightarrow \infty } a_n \le \lim _{n \rightarrow \infty } a^{(m)}_n = \frac{b_m}{1-{{\bar{L}}}}, \qquad \forall m \in {\mathbb {N}}. \end{aligned}$$

Since the convergent sequence $u_n$ is in particular a Cauchy sequence, $\lim _{m \rightarrow \infty } b_m = 0$ and, hence, $0 \le \lim _{n \rightarrow \infty } a_n = \limsup _{n \rightarrow \infty } a_n \le 0$. Hence, $\lim _{n \rightarrow \infty } a_n = 0$. $\square$

## Proof of Proposition 10

### Proof

Again, w.l.o.g. $d=1$. We prove the claims in the following order: Eqs. (39),  (45),  (40),  (46),  (41),  (43),  (44),  (42),  (49),  (48),  (47). The sharpness of these bounds is shown, directly after they are proved. As a start, for Eq. (39), we show that $P_{11}^{-,\infty }$ is indeed the unique fixed point of the recursion for $\{ P_{11}^{-}(nh) \}_n$ by checking that, if $P_{11}^-(nh) = \frac{1}{2} \left( \sigma ^2 h + \sqrt{4\sigma ^2 R h + \sigma ^4 h^2} \right)$, then also $P_{11}^-((n+1)h) = \frac{1}{2} \left( \sigma ^2 h + \sqrt{4\sigma ^2 R h + \sigma ^4 h^2} \right)$:

107 $$\begin{aligned} P_{11}((nh))&{\mathop {=}\limits ^{\text {eq. }(15)}} P_{11}^-(nh) \left( 1 - \frac{P_{11}^-(nh)}{P_{11}^-(nh) + R} \right) \nonumber \\&= \frac{\left( \sigma ^2 h + \sqrt{4\sigma ^2 R h + \sigma ^4 h^2} \right) R}{\sigma ^2 h + \sqrt{4\sigma ^2 R h + \sigma ^4 h^2} + 2R} , \quad \text {and} \end{aligned}$$

108 $$\begin{aligned} P_{11}^-((n+1)h)&= P_{11}(nh) + \sigma ^2 h \nonumber \\&{\mathop {=}\limits ^{\text {eq. }(107)}} \frac{1}{2} \left( \sigma ^2 h + \sqrt{4\sigma ^2 R h + \sigma ^4 h^2} \right) \nonumber \\&= P_{11}^-(nh). \end{aligned}$$

After combining Eq. (107) and Eq. (108), the recursion for $P_{11}^-$ is given by

109 $$\begin{aligned} P_{11}^-((n+1)h)&= \underbrace{\left( \frac{R}{P_{11}^-(nh) + R} \right) }_{=:\alpha (nh)} P_{11}^-(nh) + \sigma ^2 h \end{aligned}$$

110 $$\begin{aligned}&=:{{\tilde{T}}} \left( P_{11}^-(nh) \right) . \end{aligned}$$

Since *R* and $P_{11}^-(nh)$ are positive variances, we know that $\inf _{n \in [T/h + 1]} P_{11}^-(nh) \ge \sigma ^2 h$, and hence $\max _{n \in [T/h + 1]} \alpha (nh) \le R / (\sigma ^2 h + R) < 1$. Hence, ${{\tilde{T}}}$ is a contraction. By BFT, $P_{11}^{-,\infty }$ is the unique (attractive) fixed point of ${{\tilde{T}}}$, and the sequence $\{ \vert P_{11}^{-}(nh) - P_{11}^{-,\infty } \vert \}_n$ is strictly decreasing. Since, by Eqs. (15), (6) with $\theta = 0$ and Assumption 2,

111 $$\begin{aligned} P_{11}^-(h) = P_{11}(0) + \sigma ^2 h \le Kh, \end{aligned}$$

we can, using the reverse triangle inequality and the (by BFT) strictly decreasing sequence $\{ \vert P_{11}^{-}(nh) - P_{11}^{-,\infty } \vert \}_n$, derive Eq. (45):

112 $$\begin{aligned} \left| P_{11}^-(nh) \right|&\le \underbrace{\left| P_{11}^-(nh) - P_{11}^{-,\infty } \right| }_{\le \left| P_{11}^-(h) - P_{11}^{-,\infty } \right| } + \left| P_{11}^{-,\infty } \right| \end{aligned}$$

113 $$\begin{aligned}&\le \underbrace{P_{11}^-(h)}_{\le Kh} + \underbrace{2 P_{11}^{-,\infty }}_{\le Kh^{1 \wedge \frac{p+1}{2} }, \text { by } eq. (39)} \end{aligned}$$

114 $$\begin{aligned}&\le Kh^{1 \wedge \frac{p+1}{2} }, \end{aligned}$$

which is sharp because it is estimated against the maximum of the initial $P_{11}^-$ and the steady state that can both be attained. Recall that, by Eq. (107), $P_{11}(nh)$ depends continuously on $P^-_{11}(nh)$, and, hence, inserting Eq. (39) into Eq. (107) yields Eq. (40)—the necessary computation was already performed in Eq. (107). Since $P_{11}(nh)$ monotonically increases in $P_{11}^-(nh)$ (because the derivative of $P_{11}(nh)$ with respect to $P_{11}^-(nh)$ is non-negative for all $P_{11}^-(nh)$ due to $R\ge 0$; see Eq. (107)), we obtain Eq. (46):

115 $$\begin{aligned} P_{11}(nh)&{\mathop {\le }\limits ^{\text {eq. }(107)}} \frac{\left( \max _n P_{11}^-(nh) \right) R}{\max _n P_{11}^-(nh) + R} \end{aligned}$$

116 $$\begin{aligned}&{\mathop {\le }\limits ^{R \sim h^p}} \frac{Kh^{1 \wedge \frac{p+1}{2}} Kh^p}{Kh^{1 \wedge \frac{p+1}{2} } + Kh^p } \end{aligned}$$

117 $$\begin{aligned}&\le \frac{Kh^{(p+1)\wedge \frac{3p+1}{2}}}{Kh^{1 \wedge p}} \end{aligned}$$

118 $$\begin{aligned}&\le {\left\{ \begin{array}{ll} Kh^{\frac{p+1}{2}}, &{} \text{ if } p\le 1, \\ Kh^{p} , &{} \text{ if } p \ge 1, \end{array}\right. } \end{aligned}$$

119 $$\begin{aligned}&\le Kh^{p \vee \frac{p+1}{2}}, \end{aligned}$$

which is sharp because the steady state Eq. (45) has these rates. For Eq. (41), we again first construct the following recursion (from Eq. (10), Eqs. (15) and (6) with $\theta = 0$)

120 $$\begin{aligned} P^-_{01}((n+1)h)&= \underbrace{\frac{R}{P^-_{11}(nh) + R}}_{= \alpha (nh)} P^-_{01}\left( nh \right) \nonumber \\&\quad + \underbrace{\left( P_{11}(nh) + \frac{\sigma ^2 h}{2} \right) h}_{=:g(nh)} \end{aligned}$$

121 $$\begin{aligned}&= T_n\left( P^{-}_{01}(nh) \right) , \end{aligned}$$

where the $\alpha (nh)$-Lipschitz continuous contractions $T_n$ satisfy the prerequisites of Lemma 9, since $\sup _n \alpha (nh) \le R/(\sigma ^2 h + R) < 1$ (due to $\inf _n P_{11}^-(nh) \ge \sigma ^2 h$) and the sequence of fixed points $(1-\alpha (nh))^{-1}g(nh)$ of $T_n$ (defined by BFT) converges. Both $\alpha (nh)$ and *g*(*nh*) depend continuously on $P_{11}^-(nh)$. Hence, insertion of the limits Eqs. (39) and (40) yield

122 $$\begin{aligned} \lim _{n \rightarrow \infty } \left( 1 - \alpha (nh) \right) ^{-1} = \frac{\sigma ^2 h + \sqrt{4\sigma ^2 R h + \sigma ^4 h^2} + 2R}{\sigma ^2 h + \sqrt{4\sigma ^2 R h + \sigma ^4 h^2}}, \end{aligned}$$

and

123 $$\begin{aligned}&\lim _{n \rightarrow \infty } g(nh) \nonumber \\&\quad = \frac{(\sigma ^4 h^2 + (2R + \sigma ^2 h) \sqrt{4\sigma ^2 Rh + \sigma ^4 h^2} + 4R\sigma ^2 h )}{2 (\sigma ^2 h + \sqrt{4 \sigma ^2 R h + \sigma ^4 h^2} + 2R )} h. \end{aligned}$$

Now, application of Lemma 9 implies convergence of the recursion Eq. (121) to the product of these two limits Eqs. (122) and (123), i.e., Eq. (41):

$$\begin{aligned}&\lim _{n \rightarrow \infty } P_{01}^{-}(nh) \\&\quad = \lim _{n \rightarrow \infty } \left( 1 - \alpha (nh) \right) ^{-1} \times \lim _{n \rightarrow \infty } g(nh) \\&\quad = \frac{\sigma ^4 h^2 + (2R + \sigma ^2 h) \sqrt{4\sigma ^2 Rh + \sigma ^4 h^2} + 4R\sigma ^2 h }{2(\sigma ^2 h + \sqrt{4\sigma ^2 R h + \sigma ^4 h^2})} h. \end{aligned}$$

For Eqs. (43) and (44), we can simply insert Eqs. (39) and (41) for $P^-_{01}(nh)$ and $P^-_{11}(nh)$, respectively, into their definition Eq. (11):

124 $$\begin{aligned} \beta ^{\infty ,(0)}&{\mathop {=}\limits ^{\text {eq. }(11)}} \frac{P_{01}^{-,\infty }}{P_{11}^{-,\infty } + R} \end{aligned}$$

125 $$\begin{aligned}&{\mathop {=}\limits ^{\text {eqs. }(39)\hbox { and } (41)}} \frac{\sqrt{4R \sigma ^2 h + \sigma ^4 h^2}}{ \sigma ^2 h + \sqrt{4R \sigma ^2 h + \sigma ^4 h^2} } h, \end{aligned}$$

and

126 $$\begin{aligned} \beta ^{\infty ,(1)} {\mathop {=}\limits ^{\text {eqs. }(11)\hbox { and }(39)}} \frac{\sigma ^2 h + \sqrt{4\sigma ^2 R h + \sigma ^4 h^2}}{ \sigma ^2 h + \sqrt{4\sigma ^2 R h + \sigma ^4 h^2} + 2R }. \end{aligned}$$

These steady states Eqs. (43) and (44) are again unique and attractive because $\beta ^{(0)}(nh)$ and $\beta ^{(1)}(nh)$ depend continuously on $P_{11}^-(nh)$ and $P_{01}^-(nh)$. Next, recall that

127 $$\begin{aligned} P_{01}(nh)&{\mathop {=}\limits ^{\text {eq. }(15)}} \left( 1-\frac{P_{11}^{-}(nh)}{P_{11}^{-}(nh) + R} \right) P_{01}^{-}(nh) \end{aligned}$$

128 $$\begin{aligned}&= R \frac{P_{01}^{-}(nh)}{P_{11}^{-}(nh) + R} {\mathop {=}\limits ^{\text {eq. }(}} R \beta ^{(0)}(nh), \end{aligned}$$

which, since $P_{01}(nh)$ depends continuously on $\beta ^{(0)}(nh)$, implies the unique (attractive) fixed point $P^{\infty }_{01}(nh) = R \beta ^{\infty ,(0)}$, which yields Eq. (42). Now, exploiting Eq. (11) and $\inf _n P_{11}^-(nh) \ge \sigma ^2 h$ yields Eq. (49):

129 $$\begin{aligned} \left| 1 - \beta ^{(1)}(nh) \right|&= \frac{R}{P_{11}^-(nh) + R} \end{aligned}$$

130 $$\begin{aligned}&\le \frac{R}{\sigma ^2 h + R } \end{aligned}$$

131 $$\begin{aligned}&{\mathop {=}\limits ^{{R \sim h^p}}} \frac{Kh^p}{Kh + Kh^p} \end{aligned}$$

132 $$\begin{aligned}&\le Kh^{(p-1) \vee 0}, \end{aligned}$$

which is sharp because $\inf _n P_{11}^-(nh) \ge K h$ is sharp (due to Eqs. (10) and (6)). And since, for $\beta ^{(0)}$, maximizing over both $P_{01}^-(nh)$ and $P_{11}^-(nh)$ at the same time does not yield a sharp bound (while above in Eqs. (129) and (119) the maximization over just one quantity does), we prove Eq. (48) by inductively showing that

133 $$\begin{aligned}&\left| \beta ^{(0)}(nh) \right| \le {{\hat{\beta }}} h, \qquad \forall n \in {\mathbb {N}}, \end{aligned}$$

134 $$\begin{aligned}&\text {with} \qquad {{\hat{\beta }}} :=\left( \frac{2K_0}{\sigma ^2} + \frac{1}{2} \right) \vee 1>0, \end{aligned}$$

where $K_0 > 0$ is the constant from Assumption 2. The constant ${{\hat{\beta }}}$ is independent of *n* and a possible choice for *K* in Eq. (48).

The base case ($n=1$) follows from

135 $$\begin{aligned} \left| \beta ^{(0)}(h) \right|&= \frac{\left| P_{01}^-(h) \right| }{P_{11}^-(h) + R} \end{aligned}$$

136 $$\begin{aligned}&{\mathop {\le }\limits ^{\text {eq. }(10)}} \frac{ \left| P_{01}(0) \right| + h P_{11}(0) + \frac{\sigma ^2}{2} h^2 }{\sigma ^2 h} \end{aligned}$$

137 $$\begin{aligned}&{\mathop {\le }\limits ^{\text {Ass. }2}} \left( \frac{2K_0}{\sigma ^2} + \frac{1}{2} \right) h \end{aligned}$$

138 $$\begin{aligned}&\le {{\hat{\beta }}} h. \end{aligned}$$

In the following inductive step ($n-1 \rightarrow n$) we, to avoid notational clutter, simply denote $P^-((n-1)h)_{ij}$ by $P^-_{ij}$ which leaves us—by Eq. (11), (10) and (15)—with the following term to bound:

139 $$\begin{aligned} \left| \beta ^{(0)}(nh) \right|&= \frac{\left| P^-_{01}(nh) \right| }{P^-_{11}(nh) + R} \end{aligned}$$

140 $$\begin{aligned}&\le \frac{\left| P^-_{01} \right| \alpha (nh) + hP^-_{11} \alpha (nh) + \frac{\sigma ^2}{2} h^2}{P^-_{11}\alpha (nh) + \sigma ^2 h + R}, \end{aligned}$$

with $\alpha (nh) = \left( 1 - \frac{P^-_{11}}{P^-_{11}+R} \right) = \frac{R}{P^-_{11}+R}$. Application of the inductive hypothesis (i.e., $P_{01}^- \le {{\hat{\beta }}} ( P^-_{11} + R )$) yields, after some rearrangements, that

141 $$\begin{aligned} \left| \beta ^{(0)}(nh) \right|&\le \frac{{{\hat{\beta }}} \left( P^-_{11} + R \right) h \alpha (nh) + hP^-_{11} \alpha (nh) + \frac{\sigma ^2}{2} h^2}{P^-_{11}\alpha (nh) + \sigma ^2 h + R} \nonumber \\&= \frac{2 {{\hat{\beta }}} P^-_{11} R + \sigma ^2 h \left( P^-_{11} + R \right) + 2 P^-_{11} R + 2 {{\hat{\beta }}} R^2}{2\left( P^-_{11} R + \sigma ^2 h \left( P^-_{11} + R \right) + P^-_{11} R + R^2 \right) } h \nonumber \\&= \frac{2({{\hat{\beta }}} + 1)\varLambda _1 + \varLambda _2 + 2 {{\hat{\beta }}} \varLambda _3}{4\varLambda _1 + 2\varLambda _2 + 2\varLambda _3} h, \end{aligned}$$

with $$\varLambda _1 :=2 P^-_{11}R$$, $$\varLambda _2 :=\sigma ^2 h \left( P^-_{11} + R \right) $$, and $$\varLambda _3 :=R^2$$.

Now, application of ${{\hat{\beta }}} \ge 1$ yields $\vert \beta ^{(0)}(nh) \vert \le {{\hat{\beta }}} h$, which completes the inductive proof of Eq. (133). This implies Eq. (48), which is sharp because it is the order of $\beta ^{(0)}$ in the steady state Eq. (43), for all $p \in [0,\infty ]$. Now, insertion of Eq. (48) into Eq. (127) immediately yields Eq. (47), which—by Eq. (127)—inherits the sharpness of Eq. (48). $\square$

## Proof of Lemma 11

### Proof

For all $n \in [T/h + 1]$, we can estimate

142 $$\begin{aligned} \delta ^{(1)}(nh)&= \left\| m^{(1)}\left( nh\right) - f\left( m^{(0)}\left( nh\right) \right) \right\| \end{aligned}$$

143 $$\begin{aligned}&= \left\| \varPsi _{h}^{(1)}({\varvec{m}}((n-1)h) - f\left( m^{(0)}\left( nh\right) \right) \right\| \end{aligned}$$

144 $$\begin{aligned}&\le \underbrace{\left\| \varPsi _{h}^{(1)}({\varvec{m}}((n-1)h) - f\left( m^{-,(0)}\left( nh\right) \right) \right\| }_{=:J_1(h)} \nonumber \\&\quad + \underbrace{\left\| f\left( m^{-,(0)}\left( nh\right) \right) - f \left( m^{(0)} \left( nh \right) \right) \right\| }_{:=J_2(h)}, \end{aligned}$$

bound $J_1$, using the definition Eq. (14) of $\varPsi _h^{(1)}({\varvec{m}}((n-1)h)$ as well as the definition Eq. (13) of *r*(*nh*), by

145 $$\begin{aligned} J_1(h)&= \Bigg \Vert m^{{-,(1)}}(nh) - f \left( m^{{-,(0)}}(nh) \right) \end{aligned}$$

146 $$\begin{aligned}&\quad + \beta ^{(1)}(nh) \left[ f \left( m^{{-,(0)}}(nh) \right) - m^{{-,(1)}}(nh) \right] \Bigg \Vert \nonumber \\&\le \left\| 1 - \beta ^{(1)}(nh) \right\| {\left\| r(nh) \right\| } \end{aligned}$$

147 $$\begin{aligned}&{\mathop {\le }\limits ^{\text {eq. }(49)}} Kh^{(p-1)\vee 0} {\left\| r(nh) \right\| } \end{aligned}$$

and bound $J_2$, by exploiting *L*-Lipschitz continuity of *f*, inserting the definition Eq. (14) of $\varPsi ^{(0)}_h({\varvec{m}}((n-1)h)$ and applying Eq. (48) to $\left\| \beta ^{(0)}(nh) \right\|$,

148 $$\begin{aligned} J_2(h)&\le L \left\| m^{(0)}(nh) - m^{{-,(0)}}(nh) \right\| \end{aligned}$$

149 $$\begin{aligned}&\le L \left\| \beta ^{(0)}(nh) \right\| {\left\| r(nh) \right\| } \end{aligned}$$

150 $$\begin{aligned}&{\mathop {\le }\limits ^{\text {eq. }(48)}} Kh {\left\| r(nh) \right\| } . \end{aligned}$$

Altogether, after inserting these bounds into Eq. (144),

151 $$\begin{aligned}&\delta ^{(1)}(nh) \le \left( Kh^{(p-1) \vee 0} + Kh \right) {\left\| r(nh) \right\| } \end{aligned}$$

152 $$\begin{aligned}&\quad \le Kh^{((p-1)\vee 0) \wedge 1} {\left\| r(nh) \right\| } \end{aligned}$$

153 $$\begin{aligned}&\quad {\mathop {\le }\limits ^{\text {eq. }(31)}} Kh^{(p \vee 1) \wedge 2} \end{aligned}$$

154 $$\begin{aligned}&\qquad \qquad \quad + \left( Kh^{((p-1)\vee 0) \wedge 1} + Kh^{(p \vee 1) \wedge 2} \right) \delta ^{(1)}((n-1)h) \nonumber \\&\quad =:{\bar{T}} \left( \delta ^{(1)}((n-1)h) \right) . \end{aligned}$$

As $p\ge 1$ (by Assumption 4), BFT is applicable for all sufficiently small $h>0$ such that $Kh^{((p-1)\vee 0) \wedge 1} + Kh^{(p \vee 1) \wedge 2}<1$ and so ${\bar{T}}$ is a contraction with a unique fixed point $\delta ^{\infty }$ of order

155 $$\begin{aligned} \delta ^{\infty }&\le \frac{ Kh^{(p \vee 1) \wedge 2 } }{ 1 - \left( Kh^{((p-1)\vee 0) \wedge 1} + Kh^{(p \vee 1) \wedge 2} \right) } \end{aligned}$$

156 $$\begin{aligned}&\le Kh^{(p \vee 1) \wedge 2 }. \end{aligned}$$

We proceed with showing by induction that, for all $n \in [T/h]$,

157 $$\begin{aligned} \delta ^{(1)}(nh) \le \delta ^{(1)}(0) \vee 2\delta ^{\infty }. \end{aligned}$$

The base case $n=0$ is trivial. For the inductive step, we distinguish two cases. If $\delta ^{(1)}((n-1)h) \le \delta ^{\infty }$, then ${\bar{T}}(\delta ^{(1)}((n-1)h)) < 2 \delta ^{\infty }$, since

158 $$\begin{aligned} {\bar{T}}(\delta ^{(1)}((n-1)h)) - \delta ^{\infty }&\le \left| \delta ^{\infty } - {\bar{T}}(\delta ^{(1)}((n-1)h)) \right| \end{aligned}$$

159 $$\begin{aligned}&< \delta ^{\infty } - \underbrace{\delta ^{(1)}((n-1)h)}_{\ge 0} \end{aligned}$$

160 $$\begin{aligned}&\le \delta ^{\infty }. \end{aligned}$$

In this case,

161 $$\begin{aligned} \delta ^{(1)}(nh)&{\mathop {\le }\limits ^{\text {eq. }(154)}} {\bar{T}} \left( \delta ^{(1)}((n-1)h) \right) \end{aligned}$$

162 $$\begin{aligned}&< 2 \delta ^{\infty } \end{aligned}$$

163 $$\begin{aligned}&\le \delta ^{(1)}(0) \vee 2\delta ^{\infty }, \end{aligned}$$

where the last inequality follows from the inductive hypothesis. In the other case, namely $\delta ^{(1)}((n-1)h) > \delta ^{\infty }$, it follows that

164 $$\begin{aligned} \delta ^{(1)}(nh) - \delta ^{\infty }&{\mathop {\le }\limits ^{\text {eq. }(154)}} {\bar{T}}(\delta ^{(1)}((n-1)h)) - \delta ^{\infty } \end{aligned}$$

165 $$\begin{aligned}&\le \left| {\bar{T}}(\delta ^{(1)}((n-1)h)) - \delta ^{\infty } \right| \end{aligned}$$

166 $$\begin{aligned}&\le \left| \delta ^{(1)}((n-1)h) - \delta ^{\infty } \right| \end{aligned}$$

167 $$\begin{aligned}&= \delta ^{(1)}((n-1)h) - \delta ^{\infty }, \end{aligned}$$

which, after adding $\delta ^{\infty }$ and applying the inductive hypothesis, completes the inductive step. Hence, Eq. (157) holds. Since this bound is uniform in *n*, inserting the orders of $\delta ^{(1)}(0)$ from Lemma 7 and of $\delta ^{\infty }$ from Eq. (155) yields Eq. (50). $\square$

## Proof of Theorem 15

### Proof

Again, w.l.o.g. $d=1$. We first show that the bounds Eqs. (67) and (68) hold and then argue that they are sharp. The recursion for $P_{00}^-(nh)$ is given by

168 $$\begin{aligned} P_{00}^- ((n+1)h)&{\mathop {=}\limits ^{\text {eqs. } (10),(6)}} P_{00}(nh) + 2hP_{01}(nh) \nonumber \\&\quad + h^2 P_{11}(nh) + \frac{\sigma ^2}{3} h^3 \end{aligned}$$

169 $$\begin{aligned}&= P_{00}^-(nh) - \beta ^{(0)}(nh) P_{01}^- (nh) + \frac{\sigma ^2}{3} h^3, \nonumber \\&\quad + 2hR \beta ^{(0)}(nh) + h^2 R \beta ^{(1)}(nh) \end{aligned}$$

where we used $P_{00}(nh) = P_{00}^-(nh) - \beta ^{(0)} P_{01}^-(nh)$ and $P_{11}(nh) = R \beta ^{(1)}(nh)$ (both due to Eq. (15) and Eq. (11)), as well as $P_{01}(nh)=R \beta ^{(0)}(nh)$ (see Eq. (127)), for the last equality in Eq. (169). By $P_{01}^-(nh) \le P_{01}(nh)$ and $\vert \beta ^{(1)} \vert \le 1$ (due to Eq. (11)), application of the triangle inequality to Eq. (169) yields

170 $$\begin{aligned} P_{00}^-\left( (n+1)h \right)&\le P_{00}^- ( n h ) + \left| \beta ^{(0)}(nh) \right| \left| P_{01} (nh) \right| \nonumber \\&+ 2hR \left| \beta ^{(0)}(nh) \right| + h^2 R + \frac{\sigma ^2}{3} h^3, \end{aligned}$$

which, by Eqs. (47) and (48), implies

171 $$\begin{aligned} P_{00}^-( (n+1) h ) \le P_{00}^-(nh) + Kh^{(p+2) \wedge 3}. \end{aligned}$$

This, by $N=T/h$, implies Eq. (67). Since $P_{00}(nh) \le P_{00}^-(nh)$, this bound is also valid for $P_{00}$, i.e., Eq. (68) holds. The bound Eq. (67) is sharp, since, e.g., when the covariance matrices are in the steady state, the covariance matrix keeps growing by a rate of $Kh^{(p+2) \wedge 3}$ for all sufficiently small $h>0$, since the only negative summand in Eq. (169) is given by

172 $$\begin{aligned} \beta ^{\infty ,(0)} P_{01}^{\infty } = S_1(h) \times S_2(h) \times S_3(h) \ \in \varTheta (h^{5 \wedge \frac{3p + 7}{2} }) , \end{aligned}$$

where the factors have, due to $R \equiv Kh^p$, the following orders:

173 $$\begin{aligned} S_1(h)&= \frac{1}{2} h^2 \ \in \varTheta (h^2), \end{aligned}$$

174 $$\begin{aligned} S_2(h)&= \sqrt{(\sigma ^2 h)^2 + 4 (\sigma ^2 h) R}, \ \in \varTheta ( h^{1 \wedge \frac{p+1}{2} }), \end{aligned}$$

175 $$\begin{aligned} S_3(h)&= ((\sigma ^2 h) + 2 R) \sqrt{(\sigma ^2 h)^2 + 4 (\sigma ^2 h) R} \nonumber \\&\quad + (\sigma ^2 h)^2 + 4 (\sigma ^2 h) R \ \in \varTheta (h^{2 \wedge (p+1)}) . \end{aligned}$$

The orders in Eqs. (173) to (175) imply the order in Eq. (172). Hence, the sole negative summand $- \beta ^{\infty ,(0)} P_{01}^{\infty }$ of Eq. (169) is in $\varTheta (h^{5 \wedge \frac{3p + 7}{2} })$ and thereby of higher order than the remaining positive summands of Eq. (169):

176 $$\begin{aligned} \underbrace{2hR}_{\in \varTheta (h^{p+1})} \underbrace{\beta ^{\infty ,(0)}(nh)}_{\in \varTheta (h)}&\ \in \varTheta (h^{p+2}), \end{aligned}$$

177 $$\begin{aligned} \underbrace{h^2 R}_{\in \varTheta (h^{p+2})} \underbrace{\beta ^{\infty ,(1)}(nh)}_{\in \varTheta (1), \text { by eq. }(44)}&\ \in \varTheta (h^{p+2}), \end{aligned}$$

178 $$\begin{aligned} \frac{\sigma ^2}{3} h^3&\ \in \varTheta \left( h^{3}\right) . \end{aligned}$$

Hence, for all sufficiently small $h>0$, it still holds in the steady state that $P_{00}^-((n+1)h) - P_{00}^-(nh) \ge Kh^{(p+2) \wedge 3}$, and therefore Eq. (67) is sharp. The sharpness of Eq. (67) is inherited by Eq. (68) since, in the steady state, by Eqs. (15) and (11), $P_{00}(nh) = P_{00}^{-}(nh) - \beta ^{(0),\infty } P_{01}^{-,\infty }$ and the subtracted quantity $\beta ^{(0),\infty } P_{01}^{-,\infty }$ is—as shown above—only of order $\varTheta (h^{5 \wedge \frac{3p + 7}{2} })$. $\square$

## Footnotes

1 Here, the word ‘Bayesian’ describes the algorithm in the sense that it employs a prior over the quantity of interest and updates it by Bayes rule according to a prespecified measurement model (as also used in Skilling (1991); Chkrebtii et al. (2016); Kersting and Hennig (2016)). The ODE filter is not Bayesian in the stronger sense of Cockayne et al. (2019), and it remains an open problem to construct a Bayesian solver in this strong sense without restrictive assumptions, as discussed in Wang et al. (2018).

2 More involved correlation models of $\left\{ \left\{ {\varvec{X}}_{j}(t);\ 0 {\le } t {\le } T \right\} ;\ j \in [d] \right\}$ are straightforward to incorporate into the SDE Eq. (2), but seem complicated to analyze. Therefore, we restrict our attention to independent dimensions. See Appendix B for an explanation of this restriction. Note that one can also use a state space vector ${\varvec{X}}(t)$ which models other features of *x*(*t*) than the derivatives, as demonstrated with Fourier summands in Kersting and Mahsereci (2020).

3 According to Loscalzo and Talbot (1967), the filter might, however, suffer from numerical instability for high choices of *q*. (See Schober et al. (2019, Section 3.1) for an explanation of how such results on spline-based methods concern the ODE filter.)
